# The Exometabolome of *Xylella fastidiosa* in Contact with *Paraburkholderia phytofirmans* Supernatant Reveals Changes in Nicotinamide, Amino Acids, Biotin, and Plant Hormones

**DOI:** 10.3390/metabo14020082

**Published:** 2024-01-24

**Authors:** Oseias R. Feitosa-Junior, Andrea Lubbe, Suzanne M. Kosina, Joaquim Martins-Junior, Deibs Barbosa, Clelia Baccari, Paulo A. Zaini, Benjamin P. Bowen, Trent R. Northen, Steven E. Lindow, Aline M. da Silva

**Affiliations:** 1 Department of Biochemistry, Institute of Chemistry, University of Sao Paulo, Sao Paulo 05508-900, SP, Brazil; joaquim.junior@lnbr.cnpem.br (J.M.-J.); barbosad@iq.usp.br (D.B.); 2 The DOE Joint Genome Institute, Berkeley, CA 94720, USA; bpbowen@lbl.gov (B.P.B.); trnorthen@lbl.gov (T.R.N.); 3 Department of Plant and Microbial Biology, University of California, Berkeley, CA 94720, USA; clelia.baccari@berkeley.edu (C.B.); icelab@berkeley.edu (S.E.L.); 4 Environmental Genomics and Systems Biology Division, Lawrence Berkeley National Laboratory, Berkeley, CA 94720, USA; andrea.lubbe@brightseedbio.com (A.L.); smkosina@lbl.gov (S.M.K.); 5 Department of Plant Sciences, University of California, Davis, CA 95616, USA; pazaini@ucdavis.edu

**Keywords:** *Xylella fastidiosa*, *Paraburkholderia phytofirmans*, metabolomics, phytopathogen, liquid chromatography–mass spectrometry, MAGI

## Abstract

Microbial competition within plant tissues affects invading pathogens’ fitness. Metabolomics is a great tool for studying their biochemical interactions by identifying accumulated metabolites. *Xylella fastidiosa*, a Gram-negative bacterium causing Pierce’s disease (PD) in grapevines, secretes various virulence factors including cell wall-degrading enzymes, adhesion proteins, and quorum-sensing molecules. These factors, along with outer membrane vesicles, contribute to its pathogenicity. Previous studies demonstrated that co-inoculating *X. fastidiosa* with the *Paraburkholderia phytofirmans* strain PsJN suppressed PD symptoms. Here, we further investigated the interaction between the phytopathogen and the endophyte by analyzing the exometabolome of wild-type *X. fastidiosa* and a diffusible signaling factor (DSF) mutant lacking quorum sensing, cultivated with 20% *P. phytofirmans* spent media. Liquid chromatography–mass spectrometry (LC-MS) and the Method for Metabolite Annotation and Gene Integration (MAGI) were used to detect and map metabolites to genomes, revealing a total of 121 metabolites, of which 25 were further investigated. These metabolites potentially relate to host adaptation, virulence, and pathogenicity. Notably, this study presents the first comprehensive profile of *X. fastidiosa* in the presence of a *P. phytofirmans* spent media. The results highlight that *P. phytofirmans* and the absence of functional quorum sensing affect the ratios of glutamine to glutamate (Gln:Glu) in *X. fastidiosa*. Additionally, two compounds with plant metabolism and growth properties, 2-aminoisobutyric acid and gibberellic acid, were downregulated when *X. fastidiosa* interacted with *P. phytofirmans*. These findings suggest that *P. phytofirmans*-mediated disease suppression involves modulation of the exometabolome of *X. fastidiosa*, impacting plant immunity.

## 1. Introduction

Deciphering the molecular aspects of the interaction between *Xylella fastidiosa* and its plant hosts can provide important clues about disease development [1]. One key aspect leading to disease is its ability to modulate colonization behavior in plants and in insect vector transmission through quorum sensing (QS) mechanisms [2,3]. For this, *X. fastidiosa* uses a family of modified fatty acids known as diffusible signaling factors (DSFs) that control individual and collective behavior and expression of virulence factors [4]. Many aspects contribute to the complexity of the disease at different molecular levels. These include the bacterium’s capacity to become systemic, its ability to evade detection by the host immune system, and the subsequent overreaction of the plant immune system, causing, among other effects, plant water deprivation [1,5,6,7].

Microorganisms, including symbiotic bacteria, have the capacity to influence their host behavior by regulating the synthesis of specific compounds, thus fulfilling the metabolic and protein requirements of their host organisms [8,9,10]. Nevertheless, niche competition among microorganisms—a naturally environmental occurrence—such as a spatial dispute between a pathogen and an endophyte often alters the fitness dynamics within the host [11,12]. Nutrient competition, nutrient provision, toxin secretion, and competitor predation—which are some relational possibilities among microorganisms and their hosts—can be unveiled by metabolite-level alteration assessed by metabolomics [10,13,14,15,16].

*X. fastidiosa* causes diseases in several plant hosts of economic importance to world agriculture, including grapevines, almonds, citrus, and more recently, olive trees [1,6,17,18]. This bacterium exclusively colonizes the lumen of xylem vessels of its plant hosts and is transmitted by insect vectors like sharpshooters throughout the Americas and the spittlebug *Philaenus spumarius* in Europe [19,20,21]. *X. fastidiosa* produces biofilm and secretes several virulence factors such as cell wall-degrading enzymes (CWDEs) and lipases/esterases, among others [22,23,24]. *X. fastidiosa* does not have the type III secretion system (T3SS), which in most bacterial pathogens is responsible for the delivery of effectors/virulence factors inside host cells [25,26,27], thus reflecting the lifestyle of this phytopathogen that colonizes xylem vessels, a tissue that consists mostly of dead lignified cells [28].

*Paraburkholderia phytofirmans* strain PsJN [29], known as a grapevine endophyte, multiplies within grapevines, similarly to *X. fastidiosa* [30,31]. Co-inoculation with *X. fastidiosa* results in a significant reduction in the phytopathogenic population and disease symptoms as previously reported for other pathogens [32]. This effect involves priming innate disease resistance pathways in grapevines, leading to reduced symptoms when infected with *X. fastidiosa* [33]. Recently, in a subsequent study, *P. phytofirmans* PsJN, when topically applied with a surfactant, effectively controlled Pierce’s disease (PD) in grapevines, demonstrating systemic protection against *Xylella fastidiosa* infection, although the extent of the protection was spatially limited, with potential implications for this endophyte’s use as a biological control agent [34].

Exometabolomics (or footprinting), i.e., the analysis of secreted metabolites, is a very effective approach to tracking signatures of microorganisms, e.g., contamination signatures [35]. Integrating metabolomics and genomics helps in tracking the origins of major trends in metabolite levels in different species, but also while interacting with each other [36,37]. In this sense, the characterization of the exometabolome is a useful tool for further understanding the responses of *X. fastidiosa* to its environment and/or its interactions with other organisms. In addition, the analysis of exometabolomes has many analytical advantages over the analysis of intracellular compounds due to their lower turnover, higher stability, and, consequently, greater reproducibility of their metabolic footprint [38,39].

Here, we aimed to investigate the indirect interaction between a phytopathogen and endophyte by analyzing the exometabolome of *X. fastidiosa*, WT and Δ*rpfF*, both cultivated in a 20% *P. phytofirmans* spent medium. First, we showed that the *P. phytofirmans* spent medium was capable of disturbing *X. fastidiosa* mature biofilm formation in vitro. Additionally, we performed LC-MS analysis, revealing 121 metabolites in total, of which 25 were further investigated. Finally, using integration tools including MAGI, OmicsBox, and NetSeed, we mapped metabolites to genomes, assessed the metabolic networks, and annotated the functions of the mapped genes, respectively. This study provides further insights into the effects of QS and disease development.

## 2. Materials and Methods

### 2.1. Maintenance and Cultivation of Bacteria

The bacterial strains used in this work are listed in Table 1.

*X. fastidiosa* was grown in a PD3 medium (starch 2 g/L; Soytone 2 g/L; tryptone 4 g/L; sodium citrate 1 g/L; succinate 1 g/L; hemin chloride 10 mg/L; MgSO_4_ 7H_2_O 1 g/L; KH_2_PO_4_ 1 g/L; K_2_HPO_4_ 1.5 g/L) for 7 days. The WT and Δ*rpfF* strains were maintained in a PD3 agar medium. For Δ*rpfF*, the PD3 agar had kanamycin added to a final concentration of 50 μg/mL.

For culture in liquid medium, after 7 days of culture in PD3 agar, bacteria were transferred to 100 mL of PD3 medium and maintained at 28 °C at 100 rpm for up to 7 days. Cultures were started with OD_600nm_ = 0.05.

*P. phytofirmans* PsJN was selected from a plate of King’s B medium [44] containing rifampicin (KBR) at 100 µg/mL. Next, *P. phytofirmans* was transferred to 100 mL of PD3. *X. fastidiosa* and *P. phytofirmans* were grown at 28 °C and 100 rpm. Fresh culture was started from frozen stocks at −80 °C.

Alternatively, WT and Δ*rpfF* strains were grown in PD3 supplemented with 20% of a *P. phytofirmans* spent medium. *P. phytofirmans* PsJN grown for 1 day in PD3 medium was centrifugated at 4000× *g* for 30 min, 4 °C. The supernatant was collected, adjusted to pH 4, and subjected to vacuum filtration through a 0.22 μm membrane. The eluate was added to a regular PD3 medium to a final concentration of 20% and used for subsequent experiments.

### 2.2. X. fastidiosa Biofilm Measurement

Aliquots (2 μL, 20 μL, 200 μL, and 400 μL) of the *P. phytofirmans* PsJN spent medium eluate were added to a total 1.0 mL of PD3 liquid culture and then used to grow WT *X. fastidiosa* for 7 days at 28 °C and 200 rpm in a 24-well plate. After this period, the biofilm formed was quantified by staining with the crystal violet method, loosely, as previously described [45]. Briefly, the biofilm deposited at the air–medium interface of the WT strain was washed with distilled water, stained for 20 min with 0.1% crystal violet reagent, washed again with distilled water, and then taken up in acetone:ethanol (6:4). Quantification was performed by measuring the absorbance at 595 nm (Abs_595nm_). WT *X. fastidiosa* culture without spent medium addition grown under the same conditions was used as the control. The experiment was repeated and this time an aliquot of 500 μL of the *P. phytofirmans* PsJN spent medium eluate was added to 2.5 mL of PD3 liquid culture in glass tubes and then used to grow either WT or Δ*rpfF X. fastidiosa* strains as described before. Cultures of WT or Δ*rpfF X. fastidiosa* strains without spent medium addition grown under the same conditions were used as the control.

### 2.3. Analysis of Metabolites of the Supernatant of X. fastidiosa Cultures by Liquid Chromatography Coupled to Mass Spectrometry (LC-MS)

Samples (1 mL) of spent medium from cultures and sterile control medium were centrifuged in 1.5 mL microcentrifuge tubes at 1700× *g* for 5 min. Supernatants, containing extracellular metabolites, were lyophilized and then resuspended in LC-MS-grade methanol (300 μL). Resuspended samples were centrifuged again to pellet salts. Supernatants were dried under vacuum (Savant SpeedVac Plus SC110A, Holbrook, NY, USA) and resuspended in 500 μL of LC-MS-grade methanol containing a mixture of internal standards (25 μM of 13C-15N-L-phenylalanine, 2-amino-3-bromo-5-methylbenzoic acid, 3,6-dihydroxy-4-methylpyridazine, d4 lysine, d5-benzoic acid, and 9-anthracene carboxylic acid). The samples were filtered through a 0.22 μm microcentrifuge filtration device and transferred to 1.5 mL borosilicate glass vials (Agilent, Santa Clara, CA, USA) for LC-MS analysis as previously described [46]. Extraction blanks (blank microcentrifuge tubes taken through the entire extraction procedure to check for contaminants introduced during sample preparation) were included in the analysis. A quality control mixture (a defined mixture of common metabolites) and injection blanks (pure methanol) were also analyzed at the beginning and ends of each run to ensure no drift in retention times or signal abundances and no signs of column fouling or metabolite carryover. For polar metabolite analysis, an Agilent 1290 LC system equipped with a ZIC-pHILIC column (150 mm × 1 mm, 3.5 μm 100 Å, Merck SeQuant, Darmstadt, Germany) was used for metabolite separation with the following LC conditions: solvent A, 5 mM ammonium acetate; solvent B, 9:1 acetonitrile:H_2_O with 5 mM ammonium acetate; timetable, 0 min at 100% B, 1.5 min at 100% B, 21 min at 0% B, 27 min at 0% B, 33 min at 100% B, and 45 min at 100% B; 0.8 mL/min; column compartment temperature 40 °C. Mass spectrometry analyses were performed using a 6550 quadrupole time-of-flight mass spectrometer. Mass spectrometry data were collected at a mass range of 30–1200 *m*/*z*, drying gas rate of 11 L/min, and gas temperature of 290 °C. The nebulizer pressure was set at 30 psi and capillary voltage, at 3500 V. Data were acquired in both positive (+) and negative (−) polarities. The extracted samples were obtained in triplicate for both biological and technical replicates. Additionally, three replicate samples from the sterile control media and eleven samples from extraction blanks were processed under the same conditions. A total of 62 injections were performed, including 3–11 replicate injections.

### 2.4. LCMS Data Processing and Analysis

MassHunter qualitative analysis software (Agilent, Santa Clara, CA, USA) was used to inspect the raw data. Then, the raw data obtained were parsed using Python scripts within MetAtlas [47], which was used to extract out putative metabolite peaks using an in-house metabolite database containing *m*/*z* and retention time data [47,48]. Next, the LC-MS data were analyzed using a custom code in Perl. Each of the detected peaks was evaluated to assign a level of confidence in the identification of the compound. Compounds that were identified had a corresponding retention time and *m*/*z* for a pure standard using the same methods as above. Negative and positive polarity mode lists of compounds were retrieved and merged. A quality control analysis of retention time and intensity stability was performed using internal and external standards; based on the quality control assessment, the negative mode was selected for peak integration due to better signal and retention time stability over positive mode. In order to confirm the identity of a compound, we checked the RT difference (i.e., _abs_[RT_standard_ − RT*_µ_*_experiment_]) and the ppm error mass (i.e., [(_abs_[*m*/*z*_standard_ − *m*/*z_µ_*_experiment_])/*m*/*z*_standard_] × 10^6^) (Supplementary Table S1). Compounds with an RT difference ≤ 0.05 and ppm error mass ≤ 6 were considered to have had their identities confirmed (Supplementary Table S1) [49]. Compounds that did not pass one or both criteria (up to a ppm error mass ≤ 15) were considered putative. Next, compound abundance was normalized by strains OD in each biological replicate (Supplementary Table S2).

For further comparisons among the conditions, the compounds that satisfied the following criteria were considered expressed in the supernatants: [µ*i*_sample_ − µ*i*_blank_] − [µ*i*_medium_ − µ*i*_blank_] > 0, where µ stands for “average of peak area value” and *i* stands for any detected compound. Finally, an ANOVA test was conducted to acknowledge variability across all conditions (Supplementary Table S2). Compounds with a *p*-value ≤ 0.05 were considered statistically significant. Pairwise comparison among conditions was performed and a *t*-test with the Benjamini–Hochberg procedure at a false discovery rate (FDR) ≤ 0.05 was calculated for each compound. Compounds with statistical significance between two conditions were considered differentially expressed metabolites (DEMs) (Supplementary Table S3). Extracted ion chromatograms (EICs) were generated, one representative EIC of each extract or exometabolome was used for building a summary panel for each discussed compound, and all the remaining EICs from all biological and technical replicates were made available in the Supplementary Material (Supplementary Figures S1–S3).

A Python library for the Venn diagram was used to highlight unique and shared compounds across the conditions tested. These were referred to as *Xf* (*X. fastidiosa* WT Temecula1), Δ*rpfF* (*X. fastidiosa* Δ*rpfF*), *Xf*^sm^ (*X. fastidiosa* Temecula1 WT cultivated in PD3 medium supplemented with 20% *P. phytofirmans* spent medium), Δ*rpfF*^sm^ (*X. fastidiosa* Δ*rpfF* cultivated in PD3 medium supplemented with 20% *P. phytofirmans* spent medium), and *P. phytofirmans* PsJN (*Pp*). A matrix was built from three biological replicates of *Xf*, *Xf*^sm^, Δ*rpfF*, Δ*rpfF*^sm^, and *Pp*. This matrix was given as an input to a multivariate data analysis in a principal coordinate analysis (PCoA) [50], taking as a parameter the Euclidean distance between the samples. To compute this dataset, we used the Python library skbio and the library emperor to build the PCoA plot. Permanova and permdisp statistical analysis were made with the PCoA input data using skbio modules permanova and permdisp, respectively, and the information was added later to the PCoA plot.

A heatmap based on clustering of the compounds across conditions was generated with the Python libraries matplotlib and seaborn. The expression values of all compounds under all conditions were normalized by a z-score. A color code and symbols were added to compound names to indicate their identification level and statistical significance.

### 2.5. Integration of Metabolites and Gene Set for Functional Annotation of Exometabolomes

Whole genome annotation (in FASTA format) from *Xylella fastidiosa* Temecula1 and *Paraburkholderia phytofirmans* PsJN together with a list of InChiKey identifiers from all the compounds assigned as expressed in previous steps were given as the input for a container (Docker) of MAGI v1.0 [51,52] for metabolite and gene integration. The output spreadsheets were filtered by metabolites expressed under each given condition. Next, all genes annotated by MAGI were retrieved and duplicates were filtered out. Each gene list representing an exometabolome was submitted to the software OmicsBox 3.1 [53] for functional annotation using gene ontology. Enrichment analysis was performed, annotations with an adjusted *p*-value ≤ 0.05 were collected, and the values were log_2_ transformed and ranked. The top 5 under- and overenriched annotations from each condition were collected for comparison. The matplotlib and seaborn Python libraries were used to build a bar plot.

### 2.6. Bioinformatic Analysis

Expressed metabolites were compared in box plot and bar plot, coded by the matplotlib and seaborn Python libraries. Pairwise significance comparison was performed by the Python library statannotations and the *t*-test argument was used. When necessary, the precomputed Benjamini–Hochberg procedure with an FDR < 0.5% for pairwise comparison was used for annotations later (Supplementary Table S2).

Metabolic reactions of nicotinamide, nicotinate, and biotin were freely redrawn from annotated *X. fastidiosa* and *P. phytofirmans* Kegg pathways. Illustrations of gene operons were rebuilt based on the synteny presented in the browser IMG/DoE [54]. Mummer [55] was used for the global alignment of genomic sequences.

For the metabolome and transcriptome integration, a bubble plot was created using the matplotlib and seaborn Python libraries. The metabolites’ expression values were 2^z-score^-transformed in order to maximize expression differences and keep all values positive.

### 2.7. Cooperative Metabolic Interactions between X. fastidiosa and P. phytofirmans

In order to analyze the potential ecological microbial interactions [56] between *X. fastidiosa* and *P. phytofirmans*, a reverse ecology analysis was applied to predict the ecological structure of this symbiotic relationship [57].

For defining microbe–microbe cooperative and competitive potential in a pairwise manner, a local and customized NetSeed version [58] was used (to include the metabolic complementarity index and effective metabolic overlap index) [59,60]. The metabolic complementarity index between two species measures the ratio (range 0–1) of acquired compounds in one species that are found in the metabolic network of the other species and are not used by it. The effective metabolic overlap index of two species is the fraction of compounds required by both species and is, therefore, a measure of potential nutritional competition [59], with values ranging from 0 (no competition) to 1 (high competition).

## 3. Results

### 3.1. P. phytofirmans Interacts with X. fastidiosa through Its Exometabolome

**Figure 1:**
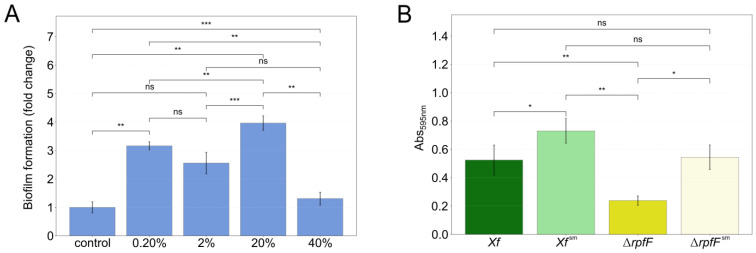
Biofilm increase in *X. fastidiosa* in response to exposure to *P. phytofirmans* secretome. (**A**) A screening of biofilm formation was performed by measurement of crystal violet staining (Abs_595nm_). WT strain was grown in a PD3 medium for 7 days with 0.2%, 2%, 20%, and 40% *v*/*v* of a 1-day PD3 *P. phytofirmans* (*Pp*) spent medium in a 24-well plate. WT strain was grown under same conditions without *P. phytofirmans* (*Pp*) spent medium and was set as a reference. (**B**) Temecula1 WT or Δ*rpfF* strains were grown in PD3 medium for 7 days with (*Xf*^sm^ and Δ*rpfF*^sm^) or without (*Xf* and Δ*rpfF*) 20% *v*/*v* of a 1-day PD3 *P. phytofirmans* (*Pp*) spent medium in a glass tube. Biofilm quantification was performed by measurement of crystal violet staining (Abs_595nm_). Error bars indicate the standard error of triplicate assays. Significantly different biofilm quantification was calculated using a *t*-test (ns: *p* ≤ 1.0 × 10^0^, *: 1.0 × 10^−2^ < *p* ≤ 5.0 × 10^−2^, **: 1.0 × 10^−3^ < *p* ≤ 1.0 × 10^−2^, ***: 1.0 × 10^−4^ < *p* ≤ 1.0 × 10^−3^).

In a prior study, Baccari and colleagues [33] demonstrated that direct contact between *X. fastidiosa* and *P. phytofirmans* was not necessary for reducing *X. fastidiosa* titer loads in xylem vessels. Here, we explore the indirect interaction between *X. fastidiosa* and *P. phytofirmans,* including the QS-insensitive mutant Δ*rpfF*. In our initial trials, we challenged *X. fastidiosa* WT Temecula1 with 0.2%, 2%, 20%, and 40% of *P. phytofirmans* spent media grown in 24-well plates (Figure 1A). Consequently, we established a specific condition for subsequent experiments. We used PD3 culture media supplemented with 20% *P. phytofirmans* PD3 spent media, which noticeably affected the *X. fastidiosa* phenotype. This impact was particularly evident in terms of biofilm intensity and deposition, as measured by the crystal violet assay (Figure 1).

During further experiments, we cultivated both *Xf* and Δ*rpfF* in regular PD3 culture media or supplemented with 20% *P. phytofirmans* PsJN spent media (*Xf*^sm^ or Δ*rpfF*^sm^). The biofilm formation of *Xf*^sm^ and Δ*rpfF*^sm^ increased by 39% and 128%, respectively, in cultivations in glass tube (Figure 1B). Moreover, a larger amount of bacterial biomass was visually observed at the medium/air interface of *Xf*^sm^ and Δ*rpfF*^sm^ compared to *Xf* and Δ*rpfF* (Figure S1). As PD3 supplemented with 20% *P. phytofirmans* spent media led to quantifiable changes in the biofilm formation profile of both bacterial strains in in vitro cultures, we hypothesized that the secreted metabolomes (exometabolomes) of *P. phytofirmans* trigger a disturbance in the exometabolomes of the tested *Xylella* strains.

### 3.2. Exometabolome Variation among X. fastidiosa Strains in Response to P. phytofirmans

**Figure 2:**
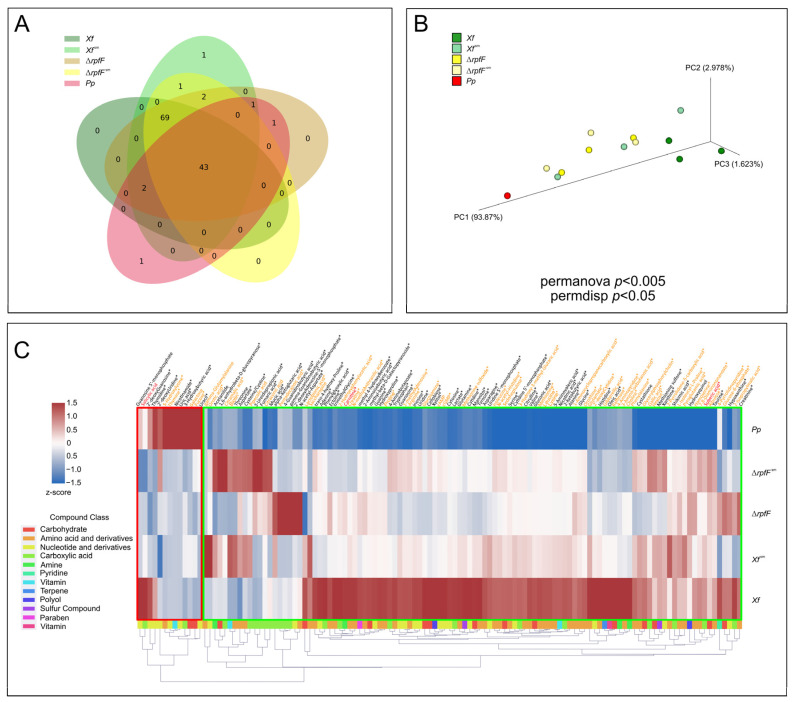
*X. fastidiosa* and *P. phytofirmans* exometabolomes. (**A**) Venn diagram of *Xf*, *Xf*^sm^, Δ*rpfF*, Δ*rpfF*^sm^, and *Pp* exometabolomes. Numbers represent shared or exclusive metabolites detected in the *Xf*, *Xf*^sm^, Δ*rpfF*, Δ*rpfF*^sm^, and *Pp* exometabolomes. (**B**) Principal coordinate analysis (PCoA) of *Xf*, *Xf*^sm^, Δ*rpfF*, Δ*rpfF*^sm^, and *Pp* exometabolomes. *Xf*, *Xf*^sm^, Δ*rpfF*, Δ*rpfF*^sm^, and *Pp* were cultured in PD3 medium for 7 days without (dark yellow and dark green circles) or with (light yellow and light green circles) 20% supernatant of *P. phytofirmans* PsJN; *Pp* was cultured in PD3 medium for 1 day (red circles). Each circle represents three technical replicates with 116 putatively identified metabolites. Permanova and permdisp statistical analyses with 999 permutations were performed, and the statistical significances are shown in the figure. (**C**) Heatmap representing the abundance of metabolites in the *Xf*, *Xf*^sm^, Δ*rpfF*, Δ*rpfF*^sm^, and *Pp* exometabolomes. Each one of the 116 compounds detected in this study is indicated in the columns and each respective strain is indicated in the rows. The color code highlights the abundance of the metabolites under the conditions tested, ranging from low (blue) and medium (white) to high (red) expression. An additional column was added with the respective compound class as stated in PubChem. Two main clusters are highlighted, in green and red boxes, as they contain the compounds highly expressed in *X. fastidiosa* and *P. phytofirmans*, respectively. A z-score normalization was performed for each compound across the different conditions. Three biological replicates of each species and treatment were used for creating this heatmap. Compounds in black were confirmed by standard run comparison (regarding RT and *m*/*z* ppm), compounds in orange had a lower level of confirmation (either or both RT and *m*/*z* ppm), and compounds in red could not be confirmed by comparison RT. Low-confidence compounds were therefore considered as putative (possibly tautomers of the compounds assigned). (*) indicates ANOVA statistical significance across conditions.

Subsequently, we investigated the exometabolomes of *Xf* and *Pp* to characterize the qualitative and quantitative signatures of their compounds. By employing an internal reference library, we were able to identify a total of 131 compounds across all strains and experimental conditions (Supplementary Tables S1–S3). Referring to the detailed criteria for detection outlined in the Section 2, we established a threshold and inferred the detection of 121 compounds among the entire spectrum of strains and conditions examined (Supplementary Table S3) [49]. Specifically, within *Xf*, *Xf*^sm^, Δ*rpfF*, Δ*rpfF*^sm^, and *Pp*, we detected 114, 119, 118, 115, and 48 compounds, respectively (Figure 2A).

There is a shared core of **43** compounds present in all five exometabolomes. Focusing exclusively on the *X. fastidiosa* strains and treatments (*Xf*, *Xf*^sm^, Δ*rpfF*, and Δ*rpfF*^sm^), the Venn diagram highlights a core of **112** compounds. Notably, the Venn diagram also unveils a distinct subset of exclusive compounds (**69**) unique to *X. fastidiosa* (*Xf*, *Xf*^sm^, Δ*rpfF*, and Δ*rpfF*^sm^). The preeminent class of compounds within the core exometabolome belongs to “nucleotides and derivatives” (comprising 32.6% of the total). Among the exclusively expressed compounds found in *X. fastidiosa strains*, amino acids and derivatives dominate (52.2%). It is noteworthy that nicotinamide stands as the sole compound exclusively identified in the *Pp* exometabolome, explored in further detail in the subsequent section.

Utilizing principal coordinate analysis (PCoA), we discerned a distinct clustering pattern within the exometabolomes, initially delineating separation based on bacterial species (*Xf* versus *Pp*), and subsequently segregating according to strains and treatments (*Xf* versus Δ*rpfF*, and *Xf*^sm^ versus Δ*rpfF*^sm^) (Figure 2B). This analysis yielded the formation of three primary and discernible clusters: (1) *Pp*, (2) *Xf*^sm^, Δ*rpfF*, and Δ*rpfF*^sm^, and (3) *Xf*. Notably, the implementation of permanova and permdisp analyses yielded statistically significant outcomes, denoted by *p*-values of <0.05 and <0.005, respectively, with respect to the distribution of samples.

Drawing from the outcomes of the PCoA, we postulate that the metabolites originating from *Pp* exert an influence on *Xf* exometabolomes, leading to a convergence in profile akin to that of the Δ*rpfF* metabolome. Interestingly, while Δ*rpfF* remains unaffected, as evidenced by the similarity to Δ*rpfF*^sm^ samples, this transformative effect is conspicuously absent. The PCoA not only highlights sample correlation but also offers a platform for constructing a working hypothesis. In essence, the assimilation of *Pp* spent media by *Xf* potentially results in a convergence of exometabolomic profiles, aligning *Xf* more closely with the characteristics of Δ*rpfF*, at least at the exometabolome level. A noteworthy observation is the similarity apparent in the sample distribution between Δ*rpfF* and Δ*rpfF*^sm^. The distribution of exometabolome samples, as represented in Figure 2B, guides our hypothesis that the interaction of *Xf* with *Pp* spent media draws it near to the phenotype of Δ*rpfF*.

Finally, we generated a heatmap illustrating the expression levels of compounds, normalized by 2^z-score^. To enhance our comprehension of the functional attributes, variability, and expression levels of the metabolites, we organized the identified compounds into groups based on PubChem classes [61]. These groups correspond to various chemical classes, including carbohydrates, amino acids (and derivatives), nucleotides (and derivatives), carboxylic acids, pyridines, amines, sulfurs, terpenes, polyols, parabens, and vitamins. Noteworthy are the compounds that serve as intermediates within metabolic pathways and the citric acid cycle (such as α-ketoglutarate, fumarate, lactate, and succinate), along with secondary metabolites (including suberic acid and shikimic acid), present in the exometabolomes of both *X. fastidiosa* and *P. phytofirmans* (Figure 2C and Supplementary Table S3).

Within *Xf*, 75 metabolites demonstrated significantly upregulated expression levels compared to *Pp*, Δ*rpfF*, *Xf*^sm^, and Δ*rpfF*^sm^. Of these, 44% were amino acids and derivatives. Similarly, *Xf*^sm^ exhibited a majority of upregulated metabolites (5), accounting for 60% of amino acids and derivatives. Conversely, Δ*rpfF* and Δ*rpfF*^sm^ displayed 8 and 12 upregulated compounds, respectively, with 50% and 41.7% being carboxylic acids. In contrast, the *Pp* exometabolome exhibited notably low metabolite detection, particularly when contrasted with those identified in *Xf* exometabolomes. Of the detected metabolites in *Pp*, 10 exhibited upregulation when compared to their counterparts in *Xf* exometabolomes, of which 50% were nucleotides or nucleotide derivatives.

The heatmap construction encompassed all compounds detected above the threshold of the blank (MeOH) and culture media (PD3 or PD3^sm^), totaling **121** compounds. Our annotation approach included two additional layers of information—statistical significance, reflecting variance among conditions, and the level of identification. The heatmap (Figure 2C) encompasses compounds that met the criteria of standard confirmation through RT and *m*/*z* ppm, and annotation also included assigning compounds demonstrating statistical significance (*p* < 0.05) based on ANOVA analysis (Supplementary Tables S2 and S3). A notable feature of the heatmap is the emergence of two distinct clusters: one primarily composed of compounds highly expressed in *Pp* and the other predominantly featuring compounds highly expressed in *Xf* strains. There exists a subset of compounds that are more highly expressed in *Xf*^sm^, Δ*rpfF*, and Δ*rpfF*^sm^ in contrast to *Xf*. Upon a comprehensive review of the overall detected metabolites, we focused the investigation on a subset of **25** compounds (amino acids, vitamins, and plant hormones), as detailed in the subsequent sections.

### 3.3. X. fastidiosa Secretes High Amounts of Amino Acids and Vitamins

First, we chose to further investigate compounds that are well known for their role in the interaction between bacteria and some species of insect hosts [62]. As such, we examined amino acid levels among the strains and conditions.

**Figure 3:**
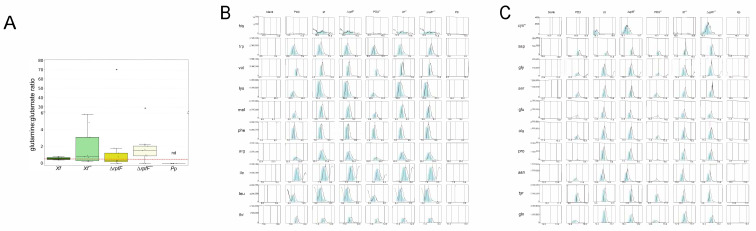
Nitrogen statuses of *Xf*, Δ*rpfF*, *Xf*^sm^, Δ*rpfF*^sm^, and *Pp* exometabolomes. (**A**) Glutamine:glutamate ratio. Average expressions of glutamine and glutamate from 3 biological replicates and 3 technical replicates were used to calculate the Gln:Glu ratio. The dotted line indicates the “0.5”, a ratio indicating cellular N-replete conditions. (**B**) EAA and (**C**) nEAA EICs of representative assessments in the blank (MeOH), PD3, PD3^sm^, *Xf*, Δ*rpfF*, *Xf*^sm^, Δ*rpfF*^sm^, and *Pp* extracts or exometabolomes.

The nutritional statuses of amino acids within the exometabolomes of *Xf*, Δ*rpfF*, *Xf^sm^,* Δ*rpfF^sm^*, and Pp were assessed, primarily utilizing the ratio of glutamine (Gln) to glutamate (Glu). This ratio, commonly employed to gauge nitrogen (N) status in various eukaryotic cells, has been linked to N limitation when <0.2 and is indicative of N-replete cells when >0.5 [10,62]. The computed median Gln:Glu ratios within the exometabolomes unveiled variations: *Xf*, Δ*rpfF*, *Xf^sm^*, and Δ*rpfF^sm^* exometabolomes all exhibited ratios predicting N-replete conditions, spanning from 0.52:1 in *Xf* to 8.33:1 in Δ*rpfF* (Figure 3A). Notably, Gln and Glu were not detected within the exometabolome of *Pp* under the assessed conditions. The 0.5 threshold for N-replete conditions was drawn in the plot, with all conditions (*Xf*, *Xf^sm^*, Δ*rpfF*, and Δ*rpfF^sm^*) surpassing this threshold—except for *Pp*, which lies below, and *Xf*, which teeters at the threshold. Importantly, our analysis did not identify any statistically significant differences between *Xf, Xf^sm^*, Δ*rpfF*, and Δ*rpfF^sm^*.

In order to provide a comprehensive understanding of compound levels within the experimental framework, we constructed a panel of EICs encompassing various controls, including the blank (MeOH) and culture media controls, PD3 and PD3^sm^ (Figure 3B,C; Supplementary Figure S1A–T). These control conditions were juxtaposed with the investigated states: *Xf*, *Xf*^sm^, Δ*rpfF*, Δ*rpfF*^sm^, and *Pp*. As anticipated, the control conditions (culture media PD3 and PD3^sm^) exhibited detectable amino acid levels. Upon scrutinizing *Xf*, *Xf*^sm^, Δ*rpfF*, and Δ*rpfF*^sm^, a noticeable pattern emerged as the following: amino acid levels, whether pertaining to nEAAs or EAAs, consistently surpass those found in blank samples and even exceed the amino acid concentrations in culture media controls. This observation aligns with the knowledge that PD3, given its protein-rich composition, inherently contains a source of amino acids. Upon closer examination, this elevated amino acid content within *Xf*, *Xf*^sm^, Δ*rpfF*, and Δ*rpfF*^sm^ challenges conventional assumptions, indicating a plausible role as an insect vector decoy within its presumed biological function. In stark contrast, the levels of EAAs and nEAAs in *Pp* are notably diminished, bordering on complete absence.

While the visual inspection of differences in the levels of essential amino acids (EAAs) and non-essential amino acids (nEAAs) does not consistently yield pronounced distinctions, we undertook individual assessments of these two amino acid categories. The nature of this evaluation displayed variability across species, strains, and treatments, with notable statistical significance emerging primarily between amino acid quantifications in *Xf* and *Xf*^sm^. Specifically, variations were evident in tyrosine, serine, proline, glutamine, aspartate, asparagine, alanine, and glycine within the nEAAs and valine, methionine, lysine, leucine, isoleucine, and arginine within the EAAs. Similarly, only histidine exhibited a discernible difference between Δ*rpfF* and Δ*rpfF*^sm^ (Figure 3B,C). Notably, within the exometabolome of *Pp*, neither EAAs nor nEAAs were detected.

Given the disparities in ionization among amino acids, merging them into a unified value for comparison across diverse conditions was not viable. Nonetheless, a consistent observation emerged as the following: essential amino acids (EAAs) exhibit higher individual abundance within *Xf*, *Xf*^sm^, Δ*rpfF*, and Δ*rpfF*^sm^ in comparison to non-essential amino acids (nEAAs).

**Figure 4:**
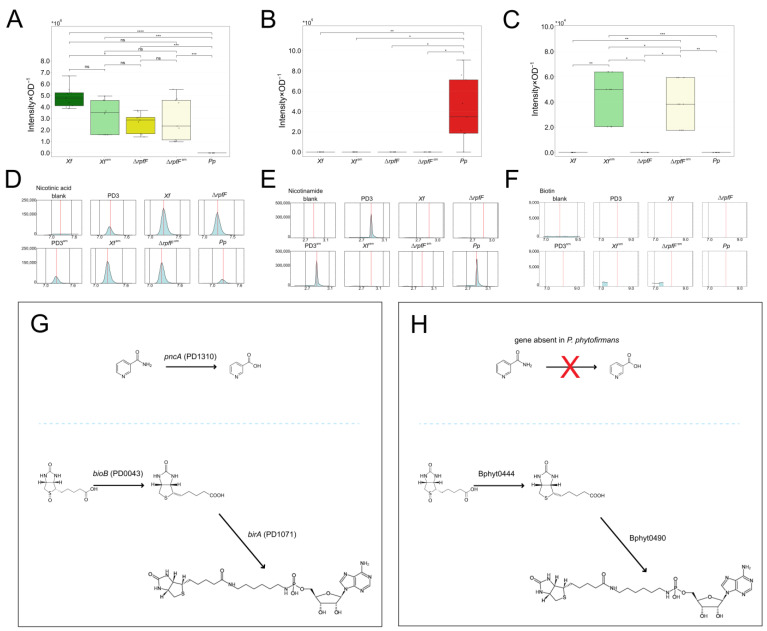
Vitamin B compound expression in *Xf*, *Xf*^sm^, Δ*rpfF*, Δ*rpfF*^sm^, and *Pp* exometabolomes. (**A**) Nicotinamide, (**B**) nicotinic acid, and (**C**) biotin expression among the exometabolomes tested. Nicotinic acid is downregulated in *Xf*^sm^ to Δ*rpfF* and Δ*rpfF*^sm^ levels. Absence of the enzyme pyrazinamidase/nicotinamidase leads to nicotinamide accumulation in *Pp* exometabolomes, but there was no detection of this compound in the *X. fastidiosa* strains. Biotin was detected only in *Xf*^sm^ and Δ*rpfF*^sm^ at very low levels. Significantly different expression by adjusted *p*-values of the pairwise comparison calculated by *t*-test (ns: *p* > 0.05, *: *p* ≤ 5.0 × 10^−2^, **: *p* ≤ 5.0 × 10^−3^, ***: *p* ≤ 5.0 × 10^−4^, ****: *p* ≤ 5.0 × 10^−5^). (**D**) Nicotinamide, (**E**) nicotinic acid, and (**F**) biotin EICs. EICs of representative assessments in the blank (MeOH), PD3, PD3^sm^, *Xf*, Δ*rpfF*, *Xf*^sm^, Δ*rpfF*^sm^, and *Pp* extracts or exometabolomes. Main reactions in “nicotinate and nicotinamide metabolism” and in “biotin metabolism” according to KEGG pathways (www.kegg.jp/pathway/) (accessed on 1 July 2023) in (**G**) *X. fastidiosa* and (**H**) *P. phytofirmans*, respectively. The letter *X* in red indicates the absence of the enzyme that converts nicotinamide into nicotinic acid.

We also investigated levels of complex B vitamins detected in the tested exometabolomes. These compounds are relevant to the relationship between an insect and host plant. Our findings illustrate the accumulation of nicotinamide in *Pp*, while it was notably absent in *Xf*, *Xf*^sm^, Δ*rpfF*, and Δ*rpfF*^sm^—this absence was evident across both normalized values (peak area intensity by OD) and EIC profiles, including in the comparison with the controls. Conversely, the presence of nicotinic acid follows an inverse pattern (Figure 4A–F, Supplementary Figure S2A–C). The product of *pncA*, namely nicotinic acid, exhibits high expression in *Xf*, along with downregulation and relatively consistent levels in *Xf*^sm^, Δ*rpfF*, and Δ*rpfF*^sm^. Biotin, on the other hand, was not detected in *Xf*, Δ*rpfF*, or *Pp*. In contrast, its presence was identified at relatively low levels in *Xf*^sm^ and Δ*rpfF*^sm^. The pathways remain conserved in both *X. fastidiosa* and *P. phytofirmans* (Figure 4G,H).

### 3.4. Two Plant Hormones Are Exclusively Secreted by X. fastidiosa

We conducted a comparative analysis of the levels of two compounds, 2-aminoisobutyric acid (AIB) and gibberellic acid (GA), detected within the exometabolomes of *Xf*, Δ*rpfF*, *Xf*^sm^, Δ*rpfF*^sm^, and *Pp*. These compounds possess implications for the interaction between *X. fastidiosa* and its plant hosts, potentially functioning as pathogenic metabolite effectors.

**Figure 5:**
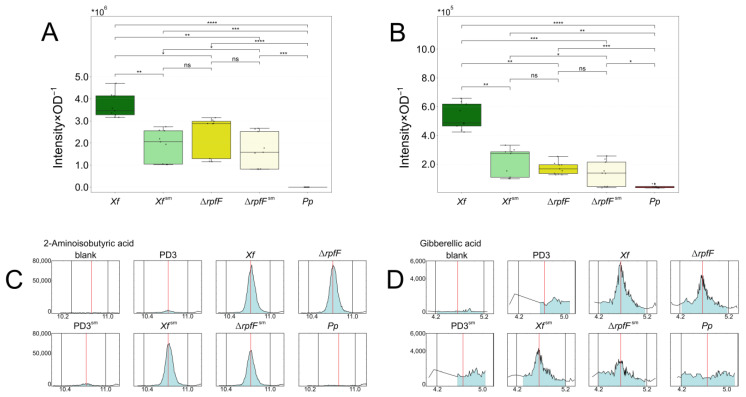
AIB and GA expression in *Xf*, *Xf*^sm^, Δ*rpfF*, Δ*rpfF*^sm^, and *Pp* exometabolomes. (**A**) AIB and (**B**) GA expression. AIB and (**B**) GA are downregulated in *Xf*^sm^ to Δ*rpfF* and Δ*rpfF*^sm^ levels. Significantly different expression by adjusted *p*-values of the pairwise comparison calculated by *t*-test (ns: *p* > 0.05, *: *p* ≤ 5.0 × 10^−2^, **: *p* ≤ 5.0 × 10^−3^, ***: *p* ≤ 5.0 × 10^−4^, ****: *p* ≤ 5.0 × 10^−5^). (**C**) AIB and (**D**) GA EICs. EICs of representative assessments in the blank (MeOH), PD3, PD3^sm^, *Xf*, Δ*rpfF*, *Xf*^sm^, Δ*rpfF*^sm^, and *Pp* extracts or exometabolomes.

Across the conditions assessed in our metabolomic analysis, AIB and GA exhibit notably similar profiles. Both compounds form three distinct clusters: (1) *Xf*, (2) *Xf*^sm^, Δ*rpfF*, and Δ*rpfF*^sm^, and (3) *Pp* (Figure 5A,B, Supplementary Figure S3A,B). Comparatively, AIB and GA levels proved significantly higher in *Xf* than in any other strain or treatment. *Xf*^sm^ displayed reduced AIB and GA expression, akin to the observed pattern in Δ*rpfF*. Conversely, Δ*rpfF*^sm^ exhibited no significant alteration in AIB and GA expression compared to Δ*rpfF*. Notably, AIB remained undetected in the *Pp* exometabolome, while GA levels ranged from 6.63% to 26.89% of those detected in *Xf*, Δ*rpfF*, *Xf*^sm^, and Δ*rpfF*^sm^ exometabolomes.

The chromatograms illustrate the contrast between the AIB levels in *Pp*, which appear lower than those in the controls, while the AIB levels in *Xf* consistently remain elevated (Figure 5C). GA was generally detected at low levels across all conditions, including *Xf*. However, both *Xf* and Δ*rpfF* exhibited levels 2 to 3 times higher than the controls. *Xf*^sm^ and Δ*rpfF*^sm^ exhibited increases of approximately 100% and 50%, respectively, compared to the culture medium controls. Notably, *Pp*’s GA levels mirror those of the controls (Figure 5C,D).

We investigated the potential synthesis of AIB and GA by *X. fastidiosa* or *P. phytofirmans* through a search for gene clusters for their synthesis. We examined the presence of homologous genes or clusters akin to the reported *aib* operon from *Rhodococcus wratislaviensis* [63] and the *Ga* operon from *Xanthomonas oryzae* pv. *oryzicola* [9,64]. While we identified orthologs of certain genes associated with AIB or GA synthesis (or metabolism) in *X. fastidiosa* or *P. phytofirmans*, we did not find them organized in operons as originally annotated in the genomes of *Rhodococcus wratislaviensis* and *Xanthomonas oryzae* pv. *oryzicola*, respectively (Supplementary Tables S14 and S15).

### 3.5. Exometabolome, Genome, and Transcriptome Integration for X. fastidiosa and P. phytofirmans

Firstly, we used NetSeed [58]—a tool for assessing metabolic network topology and determining a set of exogenously acquired compounds—to assess the metabolic networks of *X. fastidiosa* and *P. phytofirmans*, predicting them as profiles in cooperation or competition. The analysis indicated a low degree of complementarity (<0.32) and a moderate-to-high level of competition (>0.49) between *X. fastidiosa* and *P. phytofirmans*.

In a second approach, using MAGI v.1.0 [51], we searched for a connection between annotated genes in *X. fastidiosa* or *P. phytofirmans* genomes and the detected compounds, respectively, in *Xf*, *Xf*^sm^, Δ*rpfF*, Δ*rpfF*^sm^ or *Pp* (Table 2 and Table 3 and Supplementary Tables S4–S8). Out of the **121** identified compounds given as inputs to MAGI, **118** compounds returned results in gene-to-compound connection information from *X. fastidiosa* or *P. phytofirmans*. Specifically, all surveyed amino acids (both nEAAs and EAAs) exhibited connections with high reciprocity scores (≥2), i.e., compounds and annotated genes are both linked to the same reactions or metabolic pathways from the consulted databases RheA [65] or MetaCyc [66]. CDS coding for amino acid metabolism was previously annotated in the genomes of *X. fastidiosa* [27] and *P. phytofirmans* [43]. Results for AIB showed a high reciprocity score with 18 and 11 genes in *X. fastidiosa* and *P. phytofirmans*, respectively (Supplementary Tables S4–S7). Conversely, GA displayed a notably low reciprocity score with the only two associated genes in *X. fastidiosa* (PD0286, PD0716) and likewise with the three genes (Bphyt_2421, Bphyt_2594, and Bphyt_0368) in *P. phytofirmans* (Supplementary Table S8), suggesting a limited likelihood of synthesis by these genes and, respectively, reactions. In *P. phytofirmans*, Bphyt_5413 is linked to nicotinamide metabolism. In *X. fastidiosa*, PD0043, PD1071, and PD1494 are linked to biotin metabolism and PD0393 and PD1310, to nicotinic acid metabolism.

**Figure 6:**
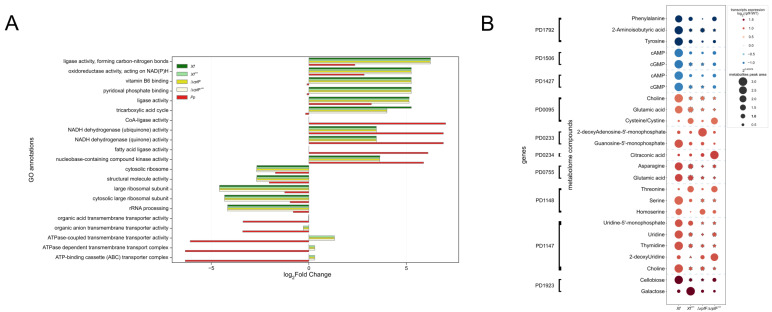
Integration of metabolomic data with functional genomics and RpfF regulon (transcriptomic). (**A**) GO enrichment analysis performed with genes retrieved from MAGI analysis. GO annotations displayed are the top 5 over- and underrepresented under the 5 conditions tested, and all GO annotations shown passed the Benjamini–Hochberg correction with an FDR < 0.5% criterion. (**B**) Public data from the RpfF regulon were crossed with genes predicted to be directly involved with the synthesis of the compounds detected in the exometabolomes. The blue-to-red color code indicates down- and upregulated genes in the RpfF regulon. The shapes and sizes of circles indicate the expression levels in peak sizes of compounds detected among the conditions tested. Peak size numbers were normalized into squared z-scores in order to maximize expression level differences among conditions. Dark gray dashed circles around dots or “bubbles” indicate DEMs in relation to *Xf*.

Next, the resulting genes from the MAGI analysis as described above were used for functional annotation via gene ontology [67,68], through OmicsBox (Supplementary Tables S9–S13). From all the annotated functions, we focused on the top five over-enriched and top five underenriched functions (Figure 6A). Despite minor variations in Δ*rpfF*, *Xf*^sm^, and Δ*rpfF*^sm^—especially when compared to *Xf*—over-represented functions generally appeared common across those conditions, such as “ligase activity, forming carbon–nitrogen bonds”, “oxidoreductase activity, acting on NAD(P)H”, and “ligase activity” (log_2_FC > 5). Functions like “vitamin B6 binding”, “pyridoxal phosphate binding”, and “tricarboxylic acid cycle” were enriched in *Xf*, Δ*rpfF*, *Xf*^sm^, and Δ*rpfF*^sm^, but underenriched in *Pp*. Conversely, “CoA-ligase activity” and “fatty acid ligase activity” were highly over-represented in *Pp* (log_2_FC > 5), whereas they were not enriched in *Xf*, Δ*rpfF*, *Xf*^sm^, or Δ*rpfF*^sm^ (Figure 6A).

Finally, we lined up our metabolome data with a previously published RpfF regulon, i.e., transcriptomic data obtained with microarray technology [3]. We did not directly and statistically integrate metabolome and transcriptome data, but, instead, we tracked genes directly linked to the detected compounds (obtained from MAGI) and cross-referenced them with the published differentially expressed genes (DEGs) in the RpfF regulon (Figure 6B). A consistent pattern exists where genes downregulated in the Δ*rpfF* strain were associated with lower levels of metabolites detected in Δ*rpfF* compared to *Xf*, including 2-aminoisobutyric acid, cAMP, cGMP, phenylalanine, and tyrosine. In contrast, the upregulated DEGs in the Δ*rpfF* strain showed no pattern at the metabolite level. Instead, metabolite expression within *Xf*^sm^, Δ*rpfF*, and Δ*rpfF*^sm^ differed when compared to *Xf*, signifying a lack of regularity but rather an upregulation of specific metabolites in these conditions. Metabolites cross-checked as upregulated in the metabolome and as a consequence of upregulated genes in the RpfF Regulon include choline, cysteine, 2-deoxyadenosine-5′-monophosphate, guanosine-5′-monophosphate, citraconic acid, asparagine, glutamic acid, threonine, serine, homoserine, uridine-5′-monophosphate, uridine, thymidine, 2-deoxyuridine, cellobiose, and galactose.

## 4. Discussion

In prior studies, it was demonstrated that the co-inoculation of *P. phytofirmans* along with *X. fastidiosa* leads to a reduction in the symptoms of leaf scorching induced by *X. fastidiosa* in grapevines. This ameliorative effect was attributed to the activation of plant defence genes, specifically involving pathways related to salicylic acid and ethylene [33]. It was further reported that the topical application of *P. phytofirmans* with a surfactant was efficient and sufficient for PD control in grapevines, even though the protection was spatially limited [34].

In our investigation, we observed that the cultivation of *X. fastidiosa* in PD3 medium supplemented with 20% spent media from *P. phytofirmans* (*Xf*^sm^ and Δ*rpfF*^sm^) resulted in a perturbation of the *X. fastidiosa* biofilm structure. In particular, the biofilm exhibits an increased volume and extends across the air–liquid medium interface, deviating from the usual compacted ring pattern observed in the liquid culture of *Xf*. A higher amount of spent media from *P. phytofirmans* (40%) and different surfaces where *X. fastidiosa* was grown (plastic versus glass) also interfered drastically in the biofilm formation (Figure 1A,B). Additionally, Baccari and colleagues [33] previously highlighted a synergistic impact arising from the concurrent infection of *X. fastidiosa* and *P. phytofirmans*, leading to the activation of plant defence mechanisms. However, in that work, there was no exploration of a direct interaction between *P. phytofirmans* and *X. fastidiosa* at the metabolomic level, which could provide insights into their relevance for conventional virulence and plant colonization.

While comparing metabolite profiles, we found a considerable disparity in the number of identified metabolites in *Pp* in comparison to *Xf*, *Xf*^sm^, Δ*rpfF*, and Δ*rpfF*^sm^. The limited abundance of detected metabolites in *Pp*, coupled with reduced variability among replicates, contributed to its distinct distribution pattern observed in the principal coordinate analysis (PCoA). While replicates of the *Xf* exometabolome exhibit close clustering, there is noticeable variability within the *Xf*^sm^, Δ*rpfF*, and Δ*rpfF*^sm^ exometabolomes, although statistical analyses such as permdisp and permanova confirmed significance across all conditions. The absence of a functional quorum-sensing (QS) system in the Δ*rpfF* strain was mirrored by its altered metabolite expression profile, akin to findings in other omic analyses such as transcriptomics and outer membrane vesicle (OMV) proteomics [3,69]. Notably, the exometabolome profile of *Xf*^sm^ shows greater similarity to that of Δ*rpfF* as opposed to *Xf*. Nevertheless, Δ*rpfF*^sm^ exometabolomes maintain an overall likeness to Δ*rpfF*. Consequently, we report that *Pp* affects the *Xf*^sm^ exometabolome, although this effect is notably diminished in the impaired DSF-producing strain (Δ*rpfF*^sm^).

*X. fastidiosa* is known to exhibit a hypersecretory trait, particularly regarding virulence proteins and extracellular vesicles (EVs), as established in prior reports [23,24,69,70]. Expanding on this, our study targeted the characterization of the *X. fastidiosa* exometabolome, focusing on low-molecular-weight compounds. Our findings indicate that, in the overall comparison among the five different groups, *Xf* has a higher expression of compounds. Pairwise comparison of *Xf* versus *Xf^sm^*, Δ*rpfF*, Δ*rpfF*^sm^, or *Pp* showed a similar pattern, with at least 80% of compounds being upregulated in *Xf*. It remains an area for further investigation to determine whether the quorum-sensing system interferes with the ability of *X. fastidiosa* to secrete metabolites. The high secretion of certain metabolites, as will be discussed below, could benefit *X. fastidiosa* from plant defence mechanisms as the phytopathogen is still not established in a higher population under the influence of the QS system [2].

Another point to highlight is that the Δ*rpfF*^sm^ exometabolome suggested a less responsive profile to *P. phytofirmans* spent media than *Xf*^sm^. Detected metabolite levels in Δ*rpfF*/Δ*rpfF*^sm^ were more similar to the levels in *Xf*/*Xf*^sm^ (e.g., they had **114** compounds detected in common, from which only two were DEM downregulated in Δ*rpfF*/Δ*rpfF*^sm^). Taken together, these observations indicate that at least partially, the detected responses to *P. phytofirmans* occur by the disturbance of DSF signaling in *Xf*. Here, we were limited to metabolites being detected through the LCMS method in a HILIC column. Other coupled MS analyses could complement our findings, e.g., using a c18 column (focused on non-polar and large compounds) or through a surface-based MS technique such as NIMS, which would increase the detected metabolites as seen for other models [71,72,73,74].

From the exometabolomes of *Xf*, *Xf*^sm^, Δ*rpfF*, Δ*rpfF*^sm^, and *Pp*, we further studied three sets of compounds: amino acids (EAAs and nEAAs), vitamin B complex, and hormones. The average Gln:Glu ratios were highly similar in *Xf* and Δ*rpfF* (0.52 and 0.57, respectively). These values (>0.5) are regarded in the literature as being indicative of N-replete cells [75]. The average Gln:Glu ratios in *Xf*^sm^ and Δ*rpfF*^sm^ showed an increased trend from treatments without contact with *P. phytofirmans* spent media, although without statistical significance. Secondly, the overall EAA and nEAA levels were inspected using their chromatogram abundance profiles. EAAs are more abundant than nEAAs in *Xf* when compared to *Xf*^sm^. It is noteworthy that a high ratio of EAAs:nEAAs was reported in the bacteriomes of four xylem-feeding insects [10]. We speculate that *X. fastidiosa* exometabolomes showing especially high EAA content might be an indication of attractiveness to insect vectors. Indeed, studies in the field that simultaneously evaluated the xylem of host plants and insects reported that adults chose plants with high amino acid concentrations [19]. The levels of EAAs and nEAAs are significantly different between *Xf* and Δ*rpfF*. Another point based on a study conducted by Daugherty and colleagues [76] indicated that the insect prefers plants with lower symptoms than plants with disease symptoms. In fact, the Δ*rpfF* mutant reaches a systemic infection faster in grapevines compared to the WT strain, and Δ*rpfF* has a lower fitness for being less transmitted by the insect vector when compared to the WT strain. Moreover, Δ*rpfF* has a restricted ability to form cell aggregates, which is the successful state of *X. fastidiosa* when acquired by the insect vector feeding on xylem sap, as reported for the WT strain [41]. In this case, it remains to be tested whether the lower level of amino acids secreted by Δ*rpfF* would add to its worse tendency to be acquired by the insect. *P. phytofirmans* had very low or totally absent detection of amino acids in its exometabolome under the conditions here tested. Nevertheless, *P. phytofirmans* has all the predicted pathways for amino acid production according to its genome annotation [43].

Similarly to amino acids, compounds within the vitamin B complex hold particular significance for insect-borne phytopathogens [77]. Our metabolomic analysis unveiled the presence of nicotinamide, nicotinic acid, and biotin. Notably, the interplay between nicotinamide and nicotinic acid mirrors the pattern of coding sequences present or absent in *X. fastidiosa* and *P. phytofirmans*. The enzyme nicotinamidase is predicted to convert the compound nicotinamide into nicotinic acid (both being isoforms of vitamin B3). In fact, in the *X. fastidiosa* genome is predicted to lie the enzyme *pncA* (nicotinamidase), whereas in the *P. phytofirmans* genome, there is no predicted homolog of a nicotinamidase gene. This conversion was indeed corroborated by our metabolomic analysis (Figure 3, Supplementary Figure S4). Symbiont bacteria, e.g., *Wolbachia* and *Baumannia*, have been reported to convert precursors and provide B vitamins to their insect xylem sap-feeding hosts [77]. Nevertheless, vitamin B3 is usually not among the B vitamins this symbiont bacteria are able to yield. It remains to be further investigated if the *X. fastidiosa* provision of this specific isoform of B vitamin increases a host plant’s attractiveness towards an insect vector. We also detected biotin, another compound from vitamin B, in *Xf*^sm^ and Δ*rpfF*^sm^. Nevertheless, there was a low level of expression in both exometabolomes and a low level of identification.

AIB, a non-proteinogenic amino acid, exists in two other isomeric forms: β-aminobutyric acid (BABA), known to induce plant disease resistance [78,79], and γ-aminobutyric acid (GABA), a neurotransmitter in animals that is also produced in plants, likely in a signaling role [80]. GABA was detected as highly abundant in the xylem sap of grapevines infected with *X. fastidiosa* [16]. AIB is reported as the immediate precursor of ethylene in higher plants. Specifically, AIB inhibits ethylene production because it acts through competitive inhibition of the conversion of 1-aminocyclopropane-1-carboxylic acid to ethylene [81]. Very little is known about AIB metabolism in bacteria, although it is reported that the AIB catabolism in *Rhodococcus wratislaviensis* C31-06 leads to its conversion into α-methyl-D-serine, and after other downstream metabolic conversions, it results in pyruvic acid [63]. In turn, gibberellic acid is a well-known plant growth promoter [82]. In bacteria, it seems the role of gibberellins is linked to pathogenicity, where secreted gibberellins can act as virulence factors, most probably by suppressing jasmonic acid formation and, ultimately, impairing the host defence response [64], as reported in the rice pathogen *Xanthomonas oryzae* pv. *oryzicola* [83]. Gibberellic acid (GA), on the other hand, functions as a phytohormone and is synthesized by both plants, fungi, and some bacterial species [84,85,86].

The identity of compounds AIB and GA was confirmed by comparing with standards by MS. Nevertheless, operons for AIB and GA synthesis are absent in *X. fastidiosa* and *P. phytofirmans* as seen in other bacteria, and it remains to be further investigated which genes are directly linked with their synthesis in *X. fastidiosa* or *P. phytofirmans*. Also, the fact that is well established that these hormones have a function in plant growth and development makes it even more interesting to address the roles of AIB and GA as virulence factors for *X. fastidiosa* during disease progression. AIB and GA do not seem to have a synergistic effect and therefore it remains unclear how their concomitant secretion would benefit *X. fastidiosa*. AIB has a higher expression than GA (3,444,408.4 intensity × OD^−1^ versus 506,103.7 intensity × OD^−1^, respectively) and in case its function prevails, it would most probably induce an impairment of host plant growth. *X. fastidiosa* could take advantage of this by a slower response from the host plant in its vasculature system. Although a GA cluster in *Burkholderia* species [87], a closely related species, has been described, such a gene cluster was not found in the *P. phytofirmans* genome (Supplementary Figure S4).

The identification and characterization of changes in specialized metabolites caused by *X. fastidiosa* in plant hosts are still in the early stages. However, collectively, these findings present a promising outlook on the reprogramming of metabolism following the interaction of plants with the phytopathogen [88]. For instance, in infected Leccino plants, higher amounts of salicylic acid were observed compared to Cellina di Nardò plants. The Leccino variety develops milder symptoms compared to those observed on the Cellina di Nardò variety [89]. It remains to be investigated if *X. fastidiosa* interacts with its plant hosts through the specialized metabolites secreted, including AIB and GA.

Reverse ecology analysis revealed generally low complementarity (<0.32) and medium-to-high competition scores (>0.49) for *X. fastidiosa* towards *P. phytofirmans* and vice versa as stated in the results section. Therefore, *X. fastidiosa* and *P. phytofirmans* are predicted competitive bacterial species. This has implications for the search for a microorganism that can act as a biocontrol for the diseases caused by *X. fastidiosa,* as seen in the natural occurrence of other endophytes, e.g., *M. mesophilicum* in orange trees [90].

Overrepresented gene ontology (GO) annotations revealed distinctions not only between *Xf* and *Pp* but also for *Xf*^sm^, Δ*rpfF*, and Δ*rpfF*^sm^. Collectively, these findings suggest that *P. phytofirmans* induces a metabolic perturbation in *Xf*^sm^, rendering it more similar to Δ*rpfF*. This observation is further supported by the overall similarity between Δ*rpfF*^sm^ and Δ*rpfF*. The specific metabolite or set of metabolites produced by *P. phytofirmans*, which is not the subject of investigation in this study and appears not to be synthesized by a Δ*rpfF* ortholog (as reported by Baccari and colleagues [33]), may still be exerting a disruptive effect on quorum sensing. Notably, quorum sensing disruption appears to be an effective strategy for disease suppression. For instance, in a study, both tobacco, a model host, and the orange tree, a natural host that produces DSF, were shown to have increased resistance to *X. fastidiosa*. The disruption in signaling confounds bacterial behavior and hinders disease development [91].

## 5. Conclusions

Overall, we demonstrated that metabolomics can be used for the prospection of *X. fastidiosa* footprinting. The secretion of EAAs and nEAAs, vitamin B complex, and the ratio between Gln:Glu are influenced by *P. phytofirmans* spent media and also, by the absence of functional QS signaling. Finally, metabolites with plant metabolism and growth properties, AIB and GA, were detected, which are downregulated in *X. fastidiosa* when in contact with *P. phytofirmans* spent media or in the absence of QS signaling. To our knowledge, this is the first report of its exometabolome in response to another endophyte.

## Figures and Tables

**Table 1:** List of bacteria and strains used in this work.

Bacteria	Strain	Original Host	Origin	Reference
*Xylella fastidiosa* subsp. *fastidiosa*	Temecula1 *Wild-Type* (WT)	*Vitis vinifera*	Temecula, California, EUA	[27,40]
	Δ*rpfF*	–	–	[41]
*Paraburkholderia phytofirmans*	PsJN	*Allium cepa*	Ontario, Canada	[42,43]

**Table 2:** Top scoring genes-to-compounds in *X. fastidiosa* according to MAGI results.

Compound Name	Gene ID	Reciprocal Score	e-Score Reaction-to-Gene	Database ID Reaction-to-Gene	e-Score Gene-to-Reaction	Database ID Gene-to-Reaction	MAGI Score
2-Aminoisobutyric acid	PD0094	2.00	200.00	ALANINE--TRNA-LIGASE-RXN	200.00	ALANINE--TRNA-LIGASE-RXN	1.58
	PD1696	2.00	200.00	RXN-16659	200.00	RXN-16659	1.58
		2.00	200.00	RXN-16649	200.00	RXN-16649	1.58
	PD1823	2.00	200.00	RHEA:20249	200.00	RHEA:20249	1.58
	PD1864	2.00	200.00	RHEA:11224	200.00	RHEA:11224	1.58
	PD1865	2.00	200.00	RHEA:23374	200.00	RHEA:23374	1.58
Alanine	PD1864	2.00	200.00	RHEA:11224	200.00	RHEA:11224	6.33
	PD1823	2.00	200.00	RHEA:20249	200.00	RHEA:20249	6.33
	PD0094	2.00	200.00	ALANINE--TRNA-LIGASE-RXN	200.00	ALANINE--TRNA-LIGASE-RXN	6.33
Arginine	PD0116	2.00	34.19	ARGININE--TRNA-LIGASE-RXN	32.31	ARGININE--TRNA-LIGASE-RXN	4.01
Asparagine	PD1947	2.00	200.00	ASPARAGINE--TRNA-LIGASE-RXN	200.00	ASPARAGINE--TRNA-LIGASE-RXN	6.33
Aspartate	PD0089	2.00	200.00	RHEA:12228	200.00	RHEA:12228	6.33
		2.00	200.00	RHEA:11375	200.00	RHEA:11375	6.33
	PD0166	2.00	200.00	RHEA:22630	200.00	RHEA:22630	6.33
	PD0291	2.00	200.00	RHEA:10932	200.00	RHEA:10932	6.33
	PD0868	2.00	200.00	RHEA:25877	200.00	RHEA:25877	6.33
	PD0946	2.00	200.00	ASPARTATE--TRNA-LIGASE-RXN	200.00	ASPARTATE--TRNA-LIGASE-RXN	6.33
	PD1273	2.00	200.00	RHEA:23777	200.00	RHEA:23777	6.33
	PD1274	2.00	200.00	RHEA:20015	200.00	RHEA:20015	6.33
	PD1627	2.00	200.00	RHEA:15753	200.00	RHEA:15753	6.33
Biotin	PD0043	2.00	140.61	2.8.1.6-RXN	145.10	2.8.1.6-RXN	5.80
	PD1071	2.00	58.42	RHEA:31118	62.94	RHEA:31118	4.66
		2.00	58.42	BIOTINLIG-RXN	62.94	BIOTINLIG-RXN	4.66
	PD1494	2.00	167.61	DETHIOBIOTIN-SYN-RXN	165.73	DETHIOBIOTIN-SYN-RXN	1.51
Cysteine	PD0655	2.00	200.00	RHEA:13285	200.00	RHEA:13285	6.33
	PD1812	2.00	156.15	RHEA:20400	154.24	RHEA:20400	5.93
	PD1841	2.00	143.27	ACSERLY-RXN	137.99	ACSERLY-RXN	5.77
	PD0118	2.00	134.13	RHEA:19398	132.24	RHEA:19398	5.71
		2.00	134.13	RHEA:25159	132.24	RHEA:25159	5.71
	PD0690	2.00	133.98	RXN0-308	132.09	RXN0-308	5.71
	PD0287	2.00	126.84	CYSTEINE--TRNA-LIGASE-RXN	124.08	CYSTEINE--TRNA-LIGASE-RXN	5.62
Gibberellic acid	PD0286	0.01	0.89	RHEA:36115	7.41	RHEA:25891	0.43
	PD0716	0.01	0.54	RHEA:36115	159.33	RHEA:13804	0.38
Nicotinic acid	PD0393	2.00	200.00	RHEA:36166	200.00	RHEA:36166	6.33
	PD1310	2.00	42.72	RHEA:14545	40.84	RHEA:14545	4.26
Glutamic acid	PD0650	2.00	200.00	RHEA:17130	200.00	RHEA:17130	6.33
	PD0654	2.00	200.00	RHEA:18052	200.00	RHEA:18052	6.33
	PD0399	2.00	200.00	RHEA:18633	200.00	RHEA:18633	6.33
	PD2062	2.00	200.00	RHEA:11613	200.00	RHEA:11613	6.33
		2.00	200.00	RHEA:15504	200.00	RHEA:15504	6.33
	PD1358	2.00	200.00	RHEA:16574	200.00	RHEA:16574	6.33
		2.00	200.00	RHEA:14329	200.00	RHEA:14329	6.33
	PD1266	2.00	200.00	RHEA:23746	200.00	RHEA:23746	6.33
	PD0089	2.00	200.00	RHEA:12228	200.00	RHEA:12228	6.33
	PD0839	2.00	200.00	RHEA:24385	200.00	RHEA:24385	6.33
	PD1447	2.00	200.00	GMP-SYN-GLUT-RXN	200.00	GMP-SYN-GLUT-RXN	6.33
	PD1848	2.00	200.00	GLURS-RXN	200.00	GLURS-RXN	6.33
	PD1026	2.00	200.00	RHEA:45804	200.00	RHEA:45804	6.33
		2.00	200.00	RHEA:16170	200.00	RHEA:16170	6.33
	PD0851	2.00	200.00	RHEA:14908	200.00	RHEA:14908	6.33
	PD0785	2.00	200.00	RHEA:15136	200.00	RHEA:15136	6.33
	PD2063	2.00	200.00	RHEA:12131	200.00	RHEA:12131	6.33
		2.00	200.00	RHEA:32192	200.00	RHEA:32192	6.33
		2.00	200.00	GLUTAMATE-SYNTHASE-FERREDOXIN-RXN	200.00	GLUTAMATE-SYNTHASE-FERREDOXIN-RXN	6.33
	PD0398	2.00	200.00	RHEA:18633	200.00	RHEA:18633	6.33
	PD0170	2.00	200.00	RHEA:21735	200.00	RHEA:21735	6.33
	PD0296	2.00	200.00	RHEA:14879	200.00	RHEA:14879	6.33
	PD0541	2.00	200.00	RHEA:15890	200.00	RHEA:15890	6.33
	PD0110	2.00	200.00	RHEA:13239	200.00	RHEA:13239	6.33
	PD0655	2.00	200.00	RHEA:13285	200.00	RHEA:13285	6.33
Glutamine	PD1447	2.00	200.00	GMP-SYN-GLUT-RXN	200.00	GMP-SYN-GLUT-RXN	6.33
	PD0584	2.00	200.00	GLUTAMINE--TRNA-LIGASE-RXN	200.00	GLUTAMINE--TRNA-LIGASE-RXN	6.33
	PD2063	2.00	200.00	GLUTAMATE-SYNTHASE-FERREDOXIN-RXN	200.00	GLUTAMATE-SYNTHASE-FERREDOXIN-RXN	6.33
Glycine	PD1750	2.00	200.00	RHEA:15482	200.00	RHEA:15482	6.33
	PD0620	2.00	200.00	GCVMULTI-RXN	200.00	GCVMULTI-RXN	6.33
		2.00	200.00	GCVP-RXN	200.00	GCVP-RXN	6.33
	PD1810	2.00	200.00	GCVMULTI-RXN	200.00	GCVMULTI-RXN	6.33
		2.00	200.00	GCVP-RXN	200.00	GCVP-RXN	6.33
	PD0827	2.00	200.00	RHEA:17453	200.00	RHEA:17453	6.33
	PD0704	2.00	200.00	RHEA:19938	200.00	RHEA:19938	6.33
	PD0844	2.00	200.00	RHEA:13557	200.00	RHEA:13557	6.33
	PD0773	2.00	200.00	RXN0-7068	200.00	RXN0-7068	6.33
		2.00	200.00	RXN0-7082	200.00	RXN0-7082	6.33
	PD0841	2.00	170.04	GLYCINE--TRNA-LIGASE-RXN	168.01	GLYCINE--TRNA-LIGASE-RXN	6.06
	PD0840	2.00	164.75	GLYCINE--TRNA-LIGASE-RXN	162.87	GLYCINE--TRNA-LIGASE-RXN	6.02
Histidine	PD1267	2.00	200.00	RHEA:20641	200.00	RHEA:20641	6.33
	PD1772	2.00	60.65	RHEA:20641	58.79	RHEA:20641	4.66
	PD1270	2.00	26.76	HISTIDINE--TRNA-LIGASE-RXN	24.84	HISTIDINE--TRNA-LIGASE-RXN	3.76
Isoleucine	PD1437	2.00	200.00	ISOLEUCINE--TRNA-LIGASE-RXN	200.00	ISOLEUCINE--TRNA-LIGASE-RXN	6.33
Leucine	PD1230	2.00	200.00	LEUCINE--TRNA-LIGASE-RXN	200.00	LEUCINE--TRNA-LIGASE-RXN	6.33
Lysine	PD0404	2.00	200.00	LYSINE--TRNA-LIGASE-RXN	200.00	LYSINE--TRNA-LIGASE-RXN	6.33
	PD1514	2.00	69.38	RXN-1961	78.45	RXN-1961	4.86
	PD2000	2.00	55.90	RHEA:15944	53.31	RHEA:15944	1.14
Methionine	PD1590	2.00	200.00	METHIONINE--TRNA-LIGASE-RXN	200.00	METHIONINE--TRNA-LIGASE-RXN	6.33
		2.00	200.00	RXN-16165	200.00	RXN-16165	6.33
Phenylalanine	PD1911	2.00	200.00	PHENYLALANINE--TRNA-LIGASE-RXN	200.00	PHENYLALANINE--TRNA-LIGASE-RXN	6.33
	PD0665	2.00	200.00	RXN-15898	200.00	RXN-15898	1.58
Proline	PD1635	2.00	200.00	PROLINE--TRNA-LIGASE-RXN	200.00	PROLINE--TRNA-LIGASE-RXN	6.33
Serine	PD0612	2.00	200.00	RHEA:26437	200.00	RHEA:26437	6.33
		2.00	200.00	RHEA:10532	200.00	RHEA:10532	6.33
	PD1750	2.00	200.00	RHEA:15482	200.00	RHEA:15482	6.33
	PD1318	2.00	200.00	RXN0-2161	200.00	RXN0-2161	6.33
		2.00	200.00	SERINE--TRNA-LIGASE-RXN	200.00	SERINE--TRNA-LIGASE-RXN	6.33
	PD0613	2.00	200.00	RHEA:26437	200.00	RHEA:26437	6.33
		2.00	200.00	RHEA:10532	200.00	RHEA:10532	6.33
Threonine	PD1916	2.00	200.00	THREONINE--TRNA-LIGASE-RXN	200.00	THREONINE--TRNA-LIGASE-RXN	6.33
Tryptophan	PD0612	2.00	200.00	RHEA:10532	200.00	RHEA:10532	6.33
		2.00	200.00	RHEA:26437	200.00	RHEA:26437	6.33
	PD0613	2.00	200.00	RHEA:10532	200.00	RHEA:10532	6.33
		2.00	200.00	RHEA:26437	200.00	RHEA:26437	6.33
	PD1650	2.00	47.82	TRYPTOPHAN--TRNA-LIGASE-RXN	45.82	TRYPTOPHAN--TRNA-LIGASE-RXN	4.38
Tyrosine	PD0665	2.00	200.00	RXN-15898	200.00	RXN-15898	6.33
	PD0132	2.00	33.23	TYROSINE--TRNA-LIGASE-RXN	31.35	TYROSINE--TRNA-LIGASE-RXN	3.98
Valine	PD0102	2.00	200.00	VALINE--TRNA-LIGASE-RXN	200.00	VALINE--TRNA-LIGASE-RXN	6.33

**Table 3:** Top scoring genes-to-compounds in *P. phytofirmans* according to MAGI results.

Compound Name	Gene ID	Reciprocal Score	e-Score Reaction-to-Gene	Database ID Reaction-to-Gene	e-Score Gene-to-Reaction	Database ID Gene-to-Reaction	MAGI Score
Cysteine	Bphyt_3930	2.00	200.00	RHEA:13285	200.00	RHEA:13285	6.33
	Bphyt_4072	2.00	200.00	RXN-17172	200.00	RXN-17172	6.33
	Bphyt_2579	2.00	200.00	RXN-15881	200.00	RXN-15881	6.33
		2.00	200.00	RXN0-308	200.00	RXN0-308	6.33
	Bphyt_3068	2.00	200.00	RHEA:28784	200.00	RHEA:28784	6.33
	Bphyt_0110	2.00	200.00	RHEA:13285	200.00	RHEA:13285	6.33
	Bphyt_3953	2.00	200.00	RHEA:13285	200.00	RHEA:13285	6.33
	Bphyt_2579	2.00	177.70	RXN-14385	175.79	RXN-14385	6.13
	Bphyt_2510	2.00	175.50	CYSTEINE--TRNA-LIGASE-RXN	171.77	CYSTEINE--TRNA-LIGASE-RXN	6.10
Gibberellic acid	Bphyt_5231	0.01	18.39	RXN-14318	39.82	RXN-16827	0.23
		0.01	18.39	RXN-14317	39.82	RXN-16827	0.23
		0.01	18.39	RXN-7617	39.82	RXN-16827	0.23
Nicotinamide	Bphyt_5413	2.00	200.00	RHEA:16150	200.00	RHEA:16150	6.33

## Data Availability

*X. fastidiosa* and *P. phytofirmans* genomic sequences were accessed from the GenBank RefSeq database at the National Center for Biotechnology Information (NCBI).

## Supplementary Materials

The following supporting information can be downloaded at: https://www.mdpi.com/article/10.3390/metabo14020082/s1, Figure S1: List of EICs from essential amino acids and non-essential amino acids under all conditions and replicates; Figure S2: List of EICs from nicotinic acid, nicotinamide, and biotin under all conditions and replicates; Figure S3: List of EICs from isoaminobutyric acid and gibberellic acid under all conditions and replicates; Figure S4: Comparison between *Burkholderia cepacia* genome and its close related species, *P. phytofirmans*; Table S1: Summary of the standard comparison of the metabolites; Table S2: List of metabolites with presence–absence detection analysis; Table S3: Final list of confirmed metabolites and pairwise statistical analysis; Table S4: MAGI *Xf* genome–metabolome integration; Table S5: MAGI *Xf*^sm^ genome–metabolome integration; Table S6: MAGI Δ*rpfF* genome–metabolome integration; Table S7: MAGI Δ*rpfF*^sm^ genome–metabolome integration; Table S8: MAGI *Pp* genome–metabolome integration; Table S9: *Xf* genome–metabolome extracted gene ontology enrichment; Table S10: *Xf*^sm^ genome–metabolome extracted gene ontology enrichment; Table S11: Δ*rpfF* genome–metabolome extracted gene ontology enrichment; Table S12: Δ*rpfF*^sm^ genome–metabolome extracted gene ontology enrichment; Table S13: *Pp* genome–metabolome extracted gene ontology enrichment; Table S14: AIB operon *Rhodococcus wratislaviensis* and relative homologous genes in *X. fastidiosa* and *P. phytofirmans*; Table S15: GA operon in *Xanthomonas oryzae* pv. *oryzicola* and relative homologous genes in *X. fastidiosa* and *P. phytofirmans*.

## References

[1] Sicard A., Zeilinger A.R., Vanhove M., Schartel T.E., Beal D.J., Daugherty M.P., Almeida R.P.P. (2018). *Xylella fastidiosa*: Insights into an Emerging Plant Pathogen. Annu. Rev. Phytopathol..

[2] Chatterjee S., Almeida R.P., Lindow S. (2008). Living in two worlds: The plant and insect lifestyles of *Xylella fastidiosa*. Annu. Rev. Phytopathol..

[3] Wang N., Li J.L., Lindow S.E. (2012). RpfF-dependent regulon of *Xylella fastidiosa*. Phytopathology.

[4] Ionescu M., Yokota K., Antonova E., Garcia A., Beaulieu E., Hayes T., Iavarone A.T., Lindow S.E. (2016). Promiscuous Diffusible Signal Factor Production and Responsiveness of the *Xylella fastidiosa* Rpf System. MBio.

[5] Roper C., Castro C., Ingel B. (2019). *Xylella fastidiosa*: Bacterial parasitism with hallmarks of commensalism. Curr. Opin. Plant Biol..

[6] Rapicavoli J., Ingel B., Blanco-Ulate B., Cantu D., Roper C. (2018). *Xylella fastidiosa*: An examination of a re-emerging plant pathogen. Mol. Plant Pathol..

[7] Morris C.E., Moury B. (2019). Revisiting the Concept of Host Range of Plant Pathogens. Annu. Rev. Phytopathol..

[8] Parniske M. (2018). Uptake of bacteria into living plant cells, the unifying and distinct feature of the nitrogen-fixing root nodule symbiosis. Curr. Opin. Plant Biol..

[9] Nagel R., Turrini P.C., Nett R.S., Leach J.E., Verdier V., Van Sluys M.A., Peters R.J. (2017). An operon for production of bioactive gibberellin A(4) phytohormone with wide distribution in the bacterial rice leaf streak pathogen *Xanthomonas oryzae* pv. oryzicola. New Phytol..

[10] Ankrah N.Y.D., Wilkes R.A., Zhang F.Q., Aristilde L., Douglas A.E. (2020). The Metabolome of Associations between Xylem-Feeding Insects and their Bacterial Symbionts. J. Chem. Ecol..

[11] Yang J., Masoudi A., Li H., Gu Y., Wang C., Wang M., Yu Z., Liu J. (2022). Microbial community structure and niche differentiation under different health statuses of *Pinus bungeana* in the Xiong’an New Area in China. Front. Microbiol..

[12] De Silva N.I., Brooks S., Lumyong S., Hyde K.D. (2019). Use of endophytes as biocontrol agents. Fungal Biol. Rev..

[13] Saldanha L.L., Allard P.M., Dilarri G., Codesido S., Gonzalez-Ruiz V., Queiroz E.F., Ferreira H., Wolfender J.L. (2022). Metabolomic-and Molecular Networking-Based Exploration of the Chemical Responses Induced in *Citrus sinensis* Leaves Inoculated with *Xanthomonas citri*. J. Agric. Food Chem..

[14] Ryffel F., Helfrich E.J.N., Kiefer P., Peyriga L., Portais J.C., Piel J., Vorholt J.A. (2016). Metabolic footprint of epiphytic bacteria on *Arabidopsis thaliana* leaves. ISME J..

[15] Chen X.L., Sun M.C., Chong S.L., Si J.P., Wu L.S. (2022). Transcriptomic and Metabolomic Approaches Deepen Our Knowledge of Plant-Endophyte Interactions. Front. Plant Sci..

[16] Zaini P.A., Nascimento R., Gouran H., Cantu D., Chakraborty S., Phu M., Goulart L.R., Dandekar A.M. (2018). Molecular Profiling of Pierce’s Disease Outlines the Response Circuitry of *Vitis vinifera* to *Xylella fastidiosa* Infection. Front. Plant Sci..

[17] Cariddi C., Saponari M., Boscia D., De Stradis A., Loconsole G., Nigro F., Porcelli F., Potere O., Martelli G.P. (2014). Isolation of a *Xylella fastidiosa* strain infecting olive and oleander in Apulia, Italy. J. Plant Pathol..

[18] Desprez-Loustau M.-L., Balci Y., Cornara D., Gonthier P., Robin C., Jacques M.-A. (2020). Is *Xylella fastidiosa* a serious threat to European forests?. For. Int. J. For. Res..

[19] Krugner R., Sisterson M.S., Backus E.A., Burbank L.P., Redak R.A. (2019). Sharpshooters: A review of what moves *Xylella fastidiosa*. Austral. Entomol..

[20] Saponari M., Loconsole G., Cornara D., Yokomi R.K., De Stradis A., Boscia D., Bosco D., Martelli G.P., Krugner R., Porcelli F. (2014). Infectivity and transmission of *Xylellua fastidiosa* by *Philaenus spumarius* (Hemiptera: *Aphrophoridae*) in Apulia, Italy. J. Econ. Entomol..

[21] Huang W.J., Reyes-Caldas P., Mann M., Seifbarghi S., Kahn A., Almeida R.P.P., Béven L., Heck M., Hogenhout S.A., Coaker G. (2020). Bacterial Vector-Borne Plant Diseases: Unanswered Questions and Future Directions. Molecular. Plant.

[22] Roper M.C., Greve L.C., Warren J.G., Labavitch J.M., Kirkpatrick B.C. (2007). *Xylella fastidiosa* requires polygalacturonase for colonization and pathogenicity in *Vitis vinifera* grapevines. Mol. Plant-Microbe Interact..

[23] Nascimento R., Gouran H., Chakraborty S., Gillespie H.W., Almeida-Souza H.O., Tu A., Rao B.J., Feldstein P.A., Bruening G., Goulart L.R. (2016). The Type II Secreted Lipase/Esterase LesA is a Key Virulence Factor Required for *Xylella fastidiosa* Pathogenesis in Grapevines. Sci. Rep..

[24] Feitosa O.R., Stefanello E., Zaini P.A., Nascimento R., Pierry P.M., Dandekar A.M., Lindow S.E., da Silva A.M. (2019). Proteomic and Metabolomic Analyses of *Xylella fastidiosa* OMV-Enriched Fractions Reveal Association with Virulence Factors and Signaling Molecules of the DSF Family. Phytopathology.

[25] Block A., Li G.Y., Fu Z.Q., Alfano J.R. (2008). Phytopathogen type III effector weaponry and their plant targets. Curr. Opin. Plant Biol..

[26] Kvitko B.H., Collmer A. (2023). Discovery of the Hrp Type III Secretion System in Phytopathogenic Bacteria: How Investigation of Hypersensitive Cell Death in Plants Led to a Novel Protein Injector System and a World of Inter-Organismal Molecular Interactions Within Plant Cells. Phytopathology.

[27] Van Sluys M.A., Monteiro-Vitorello C.B., Camargo L.E., Menck C.F., Da Silva A.C., Ferro J.A., Oliveira M.C., Setubal J.C., Kitajima J.P., Simpson A.J. (2002). Comparative genomic analysis of plant-associated bacteria. Annu. Rev. Phytopathol..

[28] De La Fuente L., Merfa M.V., Cobine P.A., Coleman J.J. (2022). Pathogen Adaptation to the Xylem Environment. Annu. Rev. Phytopathol..

[29] Sawana A., Adeolu M., Gupta R.S. (2014). Molecular signatures and phylogenomic analysis of the genus *Burkholderia*: Proposal for division of this genus into the emended genus *Burkholderia* containing pathogenic organisms and a new genus *Paraburkholderia* gen. nov. harboring environmental species. Front. Genet..

[30] Sessitsch A., Coenye T., Sturz A.V., Vandamme P., Barka E.A., Salles J.F., Van Elsas J.D., Faure D., Reiter B., Glick B.R. (2005). *Burkholderia phytofirmans* sp. nov., a novel plant-associated bacterium with plant-beneficial properties. Int. J. Syst. Evol. Microbiol..

[31] Mitter B., Petric A., Shin M.W., Chain P.S., Hauberg-Lotte L., Reinhold-Hurek B., Nowak J., Sessitsch A. (2013). Comparative genome analysis of *Burkholderia phytofirmans* PsJN reveals a wide spectrum of endophytic lifestyles based on interaction strategies with host plants. Front. Plant Sci..

[32] Miotto-Vilanova L., Jacquard C., Courteaux B., Wortham L., Michel J., Clement C., Barka E.A., Sanchez L. (2016). *Burkholderia phytofirmans* PsJN Confers Grapevine Resistance against *Botrytis cinerea* via a Direct Antimicrobial Effect Combined with a Better Resource Mobilization. Front. Plant Sci..

[33] Baccari C., Antonova E., Lindow S. (2019). Biological Control of Pierce’s Disease of Grape by an Endophytic Bacterium. Phytopathology.

[34] Lindow S., Koutsoukis R., Meyer K.M., Baccari C. (2023). Control of Pierce’s disease of grape with *Paraburkholderia phytofirmans* PsJN in the field. Phytopathology.

[35] Sue T., Obolonkin V., Griffiths H., Villas-Boas S.G. (2011). An Exometabolomics Approach to Monitoring Microbial Contamination in Microalgal Fermentation Processes by Using Metabolic Footprint Analysis. Appl. Environ. Microb..

[36] Drenos F. (2017). Mechanistic insights from combining genomics with metabolomics. Curr. Opin. Lipidol..

[37] Liu R., Bao Z.X., Zhao P.J., Li G.H. (2021). Advances in the Study of Metabolomics and Metabolites in Some Species Interactions. Molecules.

[38] Villas-Boas S.G., Noel S., Lane G.A., Attwood G., Cookson A. (2006). Extracellular metabolomics: A metabolic footprinting approach to assess fiber degradation in complex media. Anal. Biochem..

[39] Villas-Boas S.G., Mas S., Akesson M., Smedsgaard J., Nielsen J. (2005). Mass spectrometry in metabolome analysis. Mass. Spectrom. Rev..

[40] Newman K.L., Almeida R.P., Purcell A.H., Lindow S.E. (2003). Use of a green fluorescent strain for analysis of *Xylella fastidiosa* colonization of *Vitis vinifera*. Appl. Environ. Microbiol..

[41] Newman K.L., Almeida R.P.P., Purcell A.H., Lindow S.E. (2004). Cell-cell signaling controls *Xylella fastidiosa* interactions with both insects and plants. Proc. Natl. Acad. Sci. USA.

[42] Frommel M.I., Nowak J., Lazarovits G. (1991). Growth Enhancement and Developmental Modifications of in Vitro Grown Potato (*Solanum tuberosum* spp. *tuberosum*) as Affected by a Nonfluorescent *Pseudomonas* sp.. Plant Physiol..

[43] Weilharter A., Mitter B., Shin M.V., Chain P.S.G., Nowak J., Sessitsch A. (2011). Complete Genome Sequence of the Plant Growth-Promoting Endophyte *Burkholderia phytofirmans* Strain PsJN. J. Bacteriol..

[44] King E.O., Ward M.K., Raney D.E. (1954). Two simple media for the demonstration of pyocyanin and fluorescin. J. Lab. Clin. Med..

[45] Moll L., Badosa E., Planas M., Feliu L., Montesinos E., Bonaterra A. (2021). Antimicrobial Peptides With Antibiofilm Activity Against. Front. Microbiol..

[46] Kosina S.M., Danielewicz M.A., Mohammed M., Ray J., Suh Y., Yilmaz S., Singh A.K., Arkin A.P., Deutschbauer A.M., Northen T.R. (2016). Exometabolomics Assisted Design and Validation of Synthetic Obligate Mutualism. ACS Synth. Biol..

[47] Bowen B.P., Northen T.R. (2010). Dealing with the unknown: Metabolomics and metabolite atlases. J. Am. Soc. Mass. Spectrom..

[48] Yao Y., Sun T., Wang T., Ruebel O., Northen T., Bowen B.P. (2015). Analysis of Metabolomics Datasets with High-Performance Computing and Metabolite Atlases. Metabolites.

[49] Sumner L.W., Amberg A., Barrett D., Beale M.H., Beger R., Daykin C.A., Fan T.W.M., Fiehn O., Goodacre R., Griffin J.L. (2007). Proposed minimum reporting standards for chemical analysis. Metabolomics.

[50] Gower J.C. (1966). Some Distance Properties of Latent Root and Vector Methods Used in Multivariate Analysis. Biometrika.

[51] Erbilgin O., Rubel O., Louie K.B., Trinh M., Raad M., Wildish T., Udwary D., Hoover C., Deutsch S., Northen T.R. (2019). MAGI: A Method for Metabolite Annotation and Gene Integration. ACS Chem. Biol..

[52] Merkel D. (2014). Docker: Lightweight linux containers for consistent development and deployment. Linux J..

[53] Gotz S., Garcia-Gomez J.M., Terol J., Williams T.D., Nagaraj S.H., Nueda M.J., Robles M., Talon M., Dopazo J., Conesa A. (2008). High-throughput functional annotation and data mining with the Blast2GO suite. Nucleic Acids Res..

[54] Grigoriev I.V., Nordberg H., Shabalov I., Aerts A., Cantor M., Goodstein D., Kuo A., Minovitsky S., Nikitin R., Ohm R.A. (2012). The genome portal of the Department of Energy Joint Genome Institute. Nucleic Acids Res..

[55] Delcher A.L., Phillippy A., Carlton J., Salzberg S.L. (2002). Fast algorithms for large-scale genome alignment and comparison. Nucleic Acids Res..

[56] Michelini S., Balakrishnan B., Parolo S., Matone A., Mullaney J.A., Young W., Gasser O., Wall C., Priami C., Lombardo R. (2018). A reverse metabolic approach to weaning: In silico identification of immune-beneficial infant gut bacteria, mining their metabolism for prebiotic feeds and sourcing these feeds in the natural product space. Microbiome.

[57] Levy R., Borenstein E. (2012). Reverse Ecology: From systems to environments and back. Adv. Exp. Med. Biol..

[58] Carr R., Borenstein E. (2012). NetSeed: A network-based reverse-ecology tool for calculating the metabolic interface of an organism with its environment. Bioinformatics.

[59] Levy R., Borenstein E. (2013). Metabolic modeling of species interaction in the human microbiome elucidates community-level assembly rules. Proc. Natl. Acad. Sci. USA.

[60] Kreimer A., Doron-Faigenboim A., Borenstein E., Freilich S. (2012). NetCmpt: A network-based tool for calculating the metabolic competition between bacterial species. Bioinformatics.

[61] Kim S., Chen J., Cheng T.J., Gindulyte A., He J., He S.Q., Li Q.L., Shoemaker B.A., Thiessen P.A., Yu B. (2022). PubChem 2023 update. Nucleic Acids Res..

[62] Bennett G.M., Moran N.A. (2015). Heritable symbiosis: The advantages and perils of an evolutionary rabbit hole. Proc. Natl. Acad. Sci. USA.

[63] Hibi M., Fukuda D., Kenchu C., Nojiri M., Hara R., Takeuchi M., Aburaya S., Aoki W., Mizutani K., Yasohara Y. (2021). A three-component monooxygenase from *Rhodococcus wratislaviensis* may expand industrial applications of bacterial enzymes. Commun. Biol..

[64] Salazar-Cerezo S., Martinez-Montiel N., Garcia-Sanchez J., Perez Y.T.R., Martinez-Contreras R.D. (2018). Gibberellin biosynthesis and metabolism: A convergent route for plants, fungi and bacteria. Microbiol. Res..

[65] Bansal P., Morgat A., Axelsen K.B., Muthukrishnan V., Coudert E., Aimo L., Hyka-Nouspikel N., Gasteiger E., Kerhornou A., Neto T.B. (2022). Rhea, the reaction knowledgebase in 2022. Nucleic Acids Res..

[66] Caspi R., Billington R., Keseler I.M., Kothari A., Krummenacker M., Midford P.E., Ong W.K., Paley S., Subhraveti P., Karp P.D. (2020). The MetaCyc database of metabolic pathways and enzymes-a 2019 update. Nucleic Acids Res..

[67] Ashburner M., Ball C.A., Blake J.A., Botstein D., Butler H., Cherry J.M., Davis A.P., Dolinski K., Dwight S.S., Eppig J.T. (2000). Gene Ontology: Tool for the unification of biology. Nat. Genet..

[68] Gene Ontology C., Aleksander S.A., Balhoff J., Carbon S., Cherry J.M., Drabkin H.J., Ebert D., Feuermann M., Gaudet P., Harris N.L. (2023). The Gene Ontology knowledgebase in 2023. Genetics.

[69] Ionescu M., Zaini P.A., Baccari C., Tran S., da Silva A.M., Lindow S.E. (2014). *Xylella fastidiosa* outer membrane vesicles modulate plant colonization by blocking attachment to surfaces. Proc. Natl. Acad. Sci. USA.

[70] Smolka M.B., Martins D., Winck F.V., Santoro C.E., Castellari R.R., Ferrari F., Brum I.J., Galembeck E., Coletta H.D., Machado M.A. (2003). Proteome analysis of the plant pathogen *Xylella fastidiosa* reveals major cellular and extracellular proteins and a peculiar codon bias distribution. Proteomics.

[71] Jacoby R.P., Martyn A., Kopriva S. (2018). Exometabolomic Profiling of Bacterial Strains as Cultivated Using Arabidopsis Root Extract as the Sole Carbon Source. Mol. Plant-Microbe Interact..

[72] van Hoogstraten S.W.G., Kuik C., Arts J.J.C., Cillero-Pastor B. (2023). Molecular imaging of bacterial biofilms-a systematic review. Crit. Rev. Microbiol..

[73] Lawson C.E., Harcombe W.R., Hatzenpichler R., Lindemann S.R., Löffler F.E., O’Malley M.A., Martín H.G., Pfleger B.F., Raskin L., Venturelli O.S. (2019). Common principles and best practices for engineering microbiomes. Nat. Rev. Microbiol..

[74] Verbeeck N., Caprioli R.M., Van de Plas R. (2020). Unsupervised machine learning for exploratory data analysis in imaging mass spectrometry. Mass. Spectrom. Rev..

[75] Flynn K.J., Dickson D.M.J., Al-Amoudi O.A. (1989). The ratio of glutamine:glutamate in microalgae: A biomarker for N-status suitable for use at natural cell densities. J. Plankton Res..

[76] Daugherty M.P., Rashed A., Almeida R.P.P., Perring T.M. (2011). Vector preference for hosts differing in infection status: Sharpshooter movement and *Xylella fastidiosa* transmission. Ecol. Entomol..

[77] Douglas A.E. (2017). The B vitamin nutrition of insects: The contributions of diet, microbiome and horizontally acquired genes. Curr. Opin. Insect. Sci..

[78] Sasek V., Nováková M., Dobrev P.I., Valentová O., Burketová L. (2012). β-aminobutyric acid protects *Brassica napus* plants from infection by *Leptosphaeria maculans*. Resistance induction or a direct antifungal effect?. Eur. J. Plant Pathol..

[79] Pajot E., Le Corre D., Silué D. (2001). Phytogard^®^ and DL-β-amino Butyric Acid (BABA) Induce Resistance to Downy Mildew (*Bremia Lactucae*) in Lettuce (*Lactuca sativa L*). Eur. J. Plant Pathol..

[80] Obata K. (2013). Synaptic inhibition and γ-aminobutyric acid in the mammalian central nervous system. Proc. Jpn. Acad. B-Phys..

[81] Wang H., Zhi W., Qu H., Lin H., Jiang Y. (2015). Application of alpha-aminoisobutyric acid and beta-aminoisobutyric acid inhibits pericarp browning of harvested longan fruit. Chem. Cent. J..

[82] Phinney B.O. (1956). Growth Response of Single-Gene Dwarf Mutants in Maize to Gibberellic Acid. Proc. Natl. Acad. Sci. USA.

[83] Lu X., Hershey D.M., Wang L., Bogdanove A.J., Peters R.J. (2015). An ent-kaurene-derived diterpenoid virulence factor from *Xanthomonas oryzae* pv. *oryzicola*. New Phytol..

[84] Urbanová T., Tarkowská D., Strnad M., Hedden P. (2011). Gibberellins-Terpenoid Plant Hormones: Biological Importance and Chemical Analysis. Collect Czech. Chem. C.

[85] Tudzynski B. (2005). Gibberellin biosynthesis in fungi: Genes, enzymes, evolution, and impact on biotechnology. Appl. Microbiol. Biotechnol..

[86] Nett R.S., Montanares M., Marcassa A., Lul X., Nagel R., Charles T.C., Hedden P., Rojas M.C., Peters R.J. (2017). Elucidation of gibberellin biosynthesis in bacteria reveals convergent evolution. Nat. Chem. Biol..

[87] Joo G.J., Kang S.M., Hamayun M., Kim S.K., Na C.I., Shin D.H., Lee I.J. (2009). *Burkholderia* sp KCTC 11096BP as a newly isolated gibberellin producing bacterium. J. Microbiol..

[88] Vergine M., Nicolì F., Sabella E., Aprile A., De Bellis L., Luvisi A. (2020). Secondary Metabolites in *Xylella fastidiosa*–Plant Interaction. Pathogens.

[89] Novelli S., Gismondi A., Di Marco G., Canuti L., Nanni V., Canini A. (2019). Plant defense factors involved in *Olea europaea* resistance against *Xylella fastidiosa* infection. J. Plant Res..

[90] Azevedo J.L., Araujo W.L., Lacava P.T. (2016). The diversity of citrus endophytic bacteria and their interactions with *Xylella fastidiosa* and host plants. Genet. Mol. Biol..

[91] Caserta R., Souza-Neto R.R., Takita M.A., Lindow S., Souza A. (2017). Ectopic expression of *Xylella fastidiosa* rpfF conferring production of diffusible signal factor in transgenic tobacco and citrus alters pathogen behavior and reduces disease severity. Mol. Plant-Microbe Interact. MPMI.

