# Supplementary material for: The Exometabolome of Xylella fastidiosa in Contact with Paraburkholderia phytofirmans Supernatant Reveals Changes in Nicotinamide, Amino Acids, Biotin, and Plant Hormones
Source: Metabolites. 2024 Jan 24;14(2):82. doi: 10.3390/metabo14020082 (PMC10890622; doi:10.3390/metabo14020082)

Figure S1A. Histidine. List of EICs from Essential and Non Essential Amino Acids in all conditions and replicates.

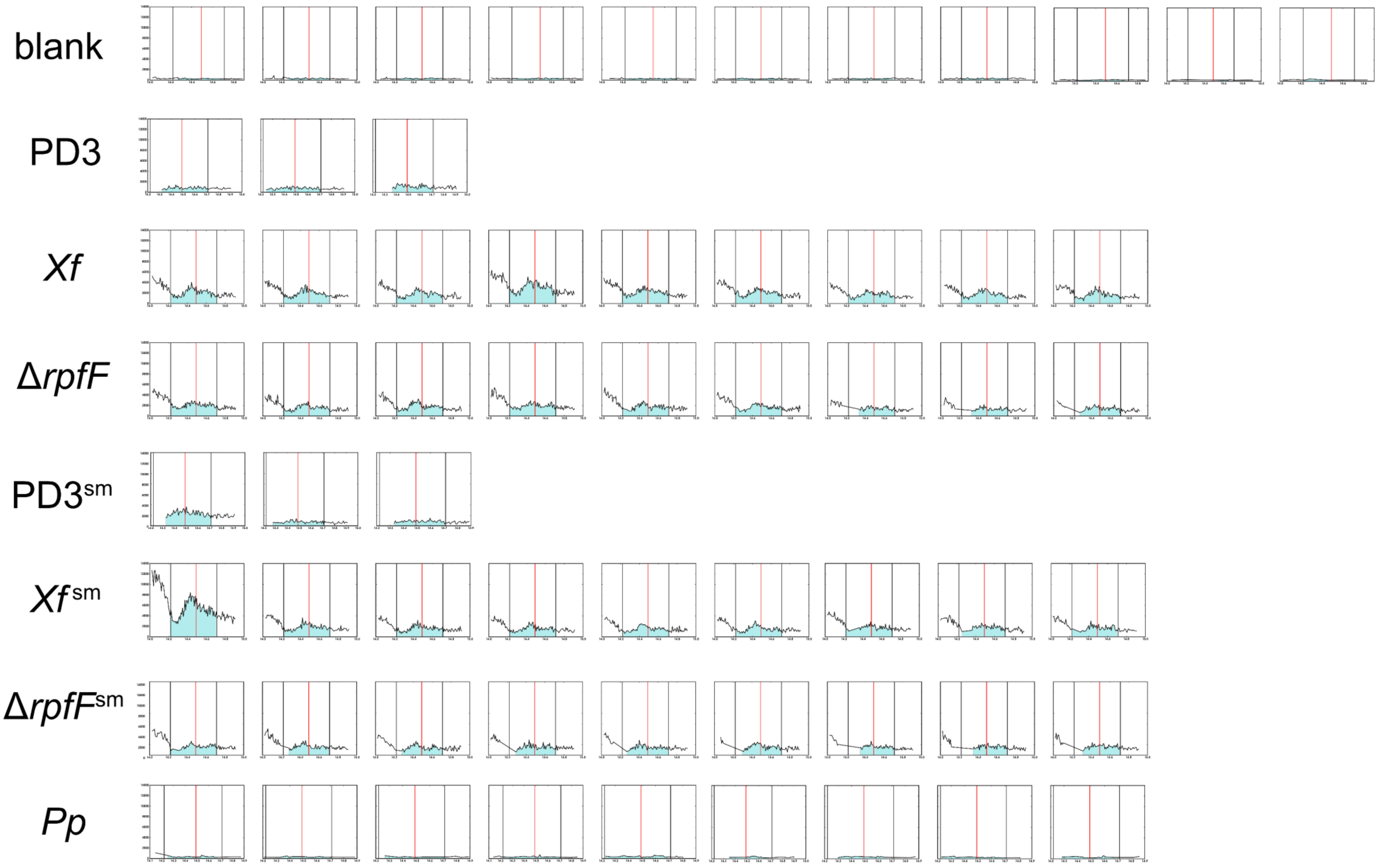

Figure S1B. Tryptophan. List of EICs from Essential and Non Essential Amino Acids in all conditions and replicates.

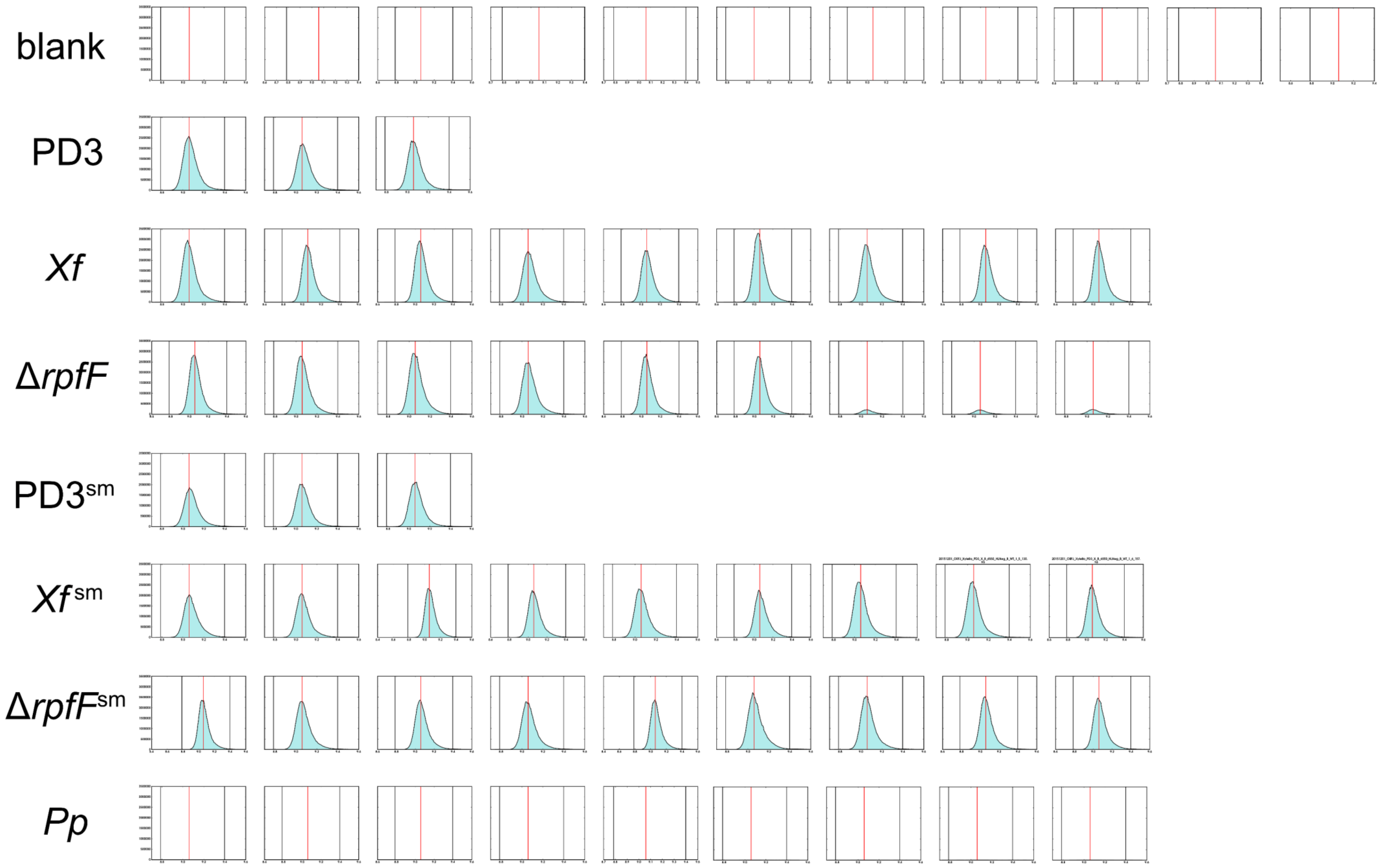

Figure S1C. Valine. List of EICs from Essential and Non Essential Amino Acids in all conditions and replicates.

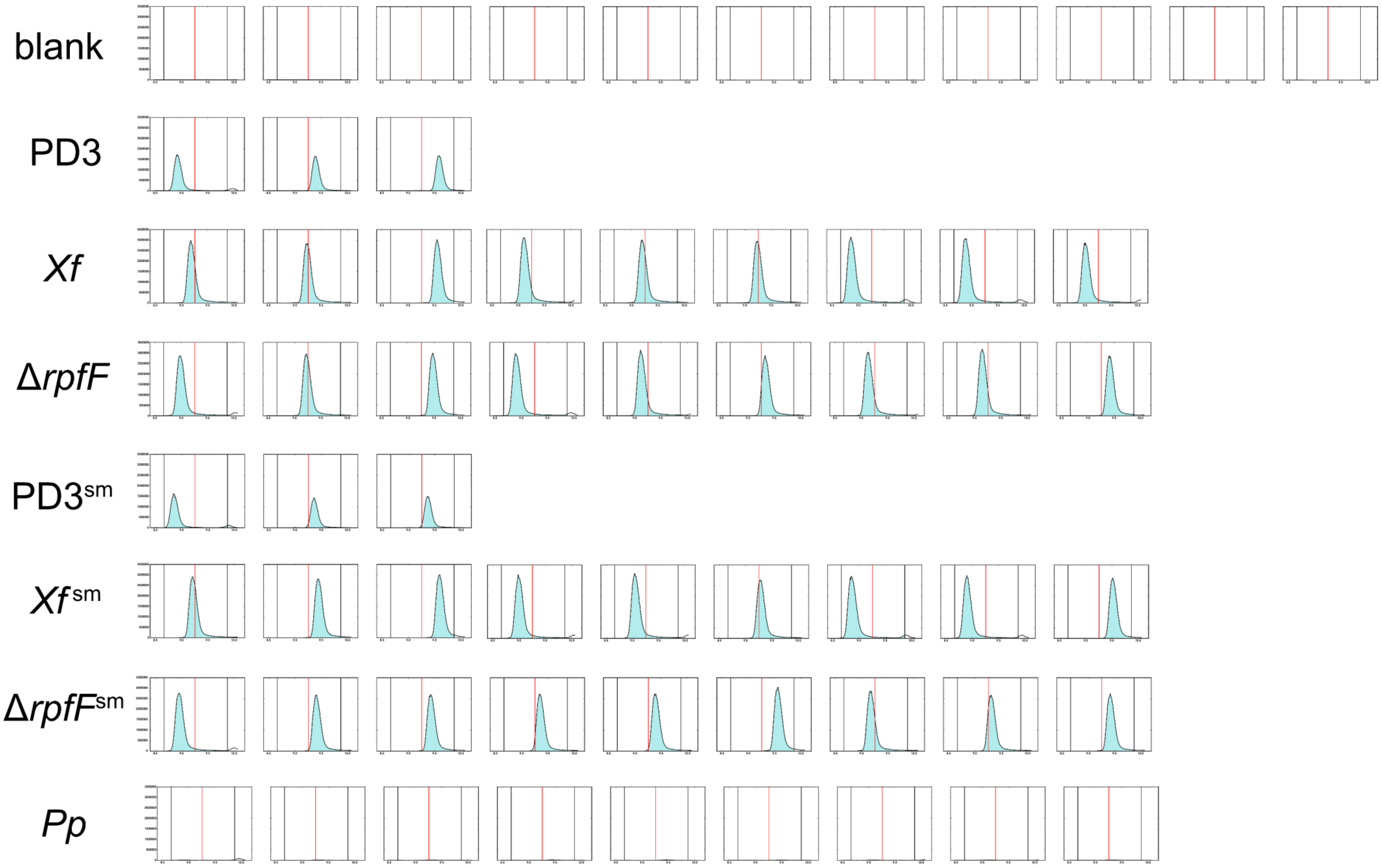

Figure S1D. Lysine. List of EICs from Essential and Non Essential Amino Acids in all conditions and replicates.

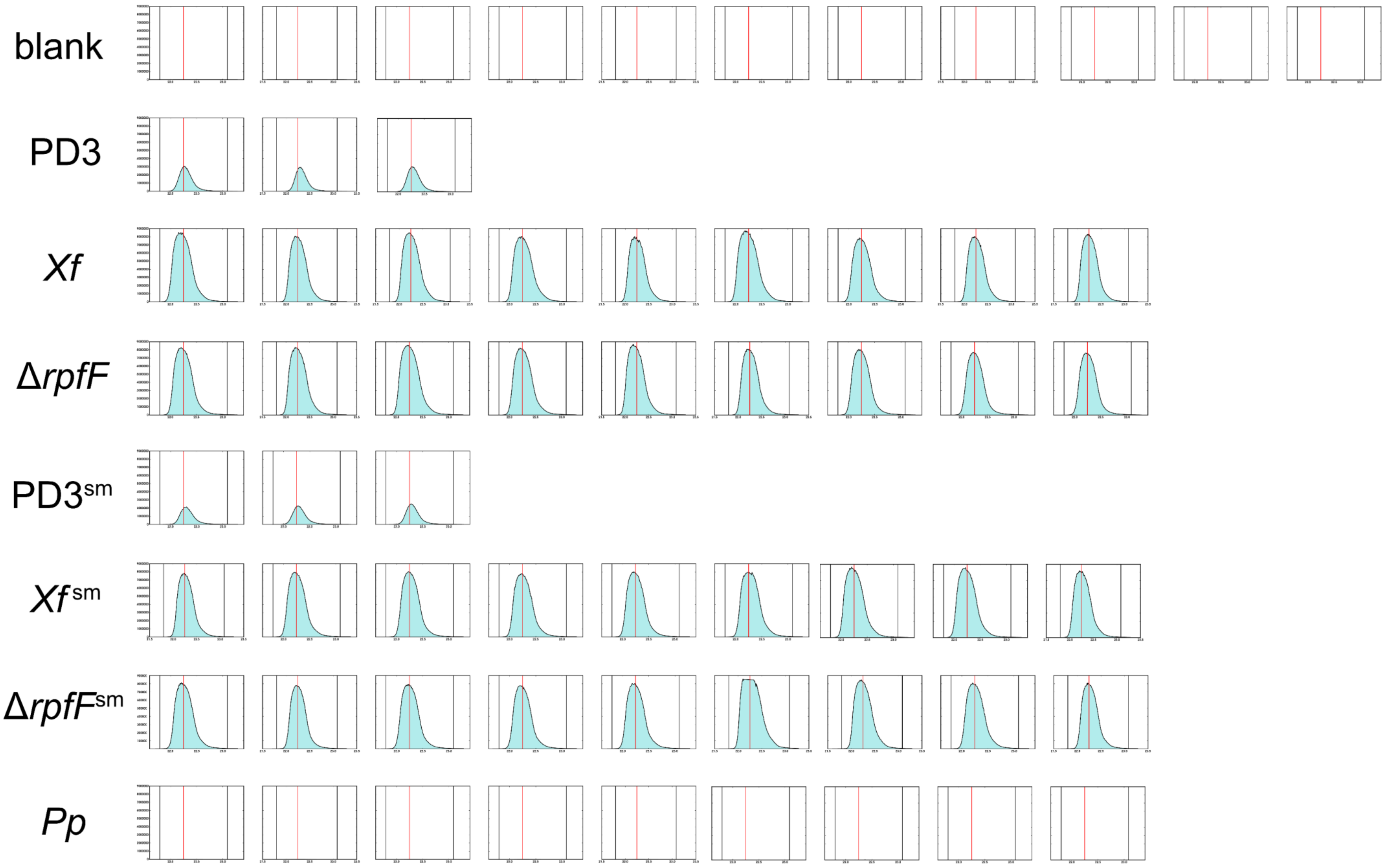

Figure S1E. Methionine. List of EICs from Essential and Non Essential Amino Acids in all conditions and replicates.

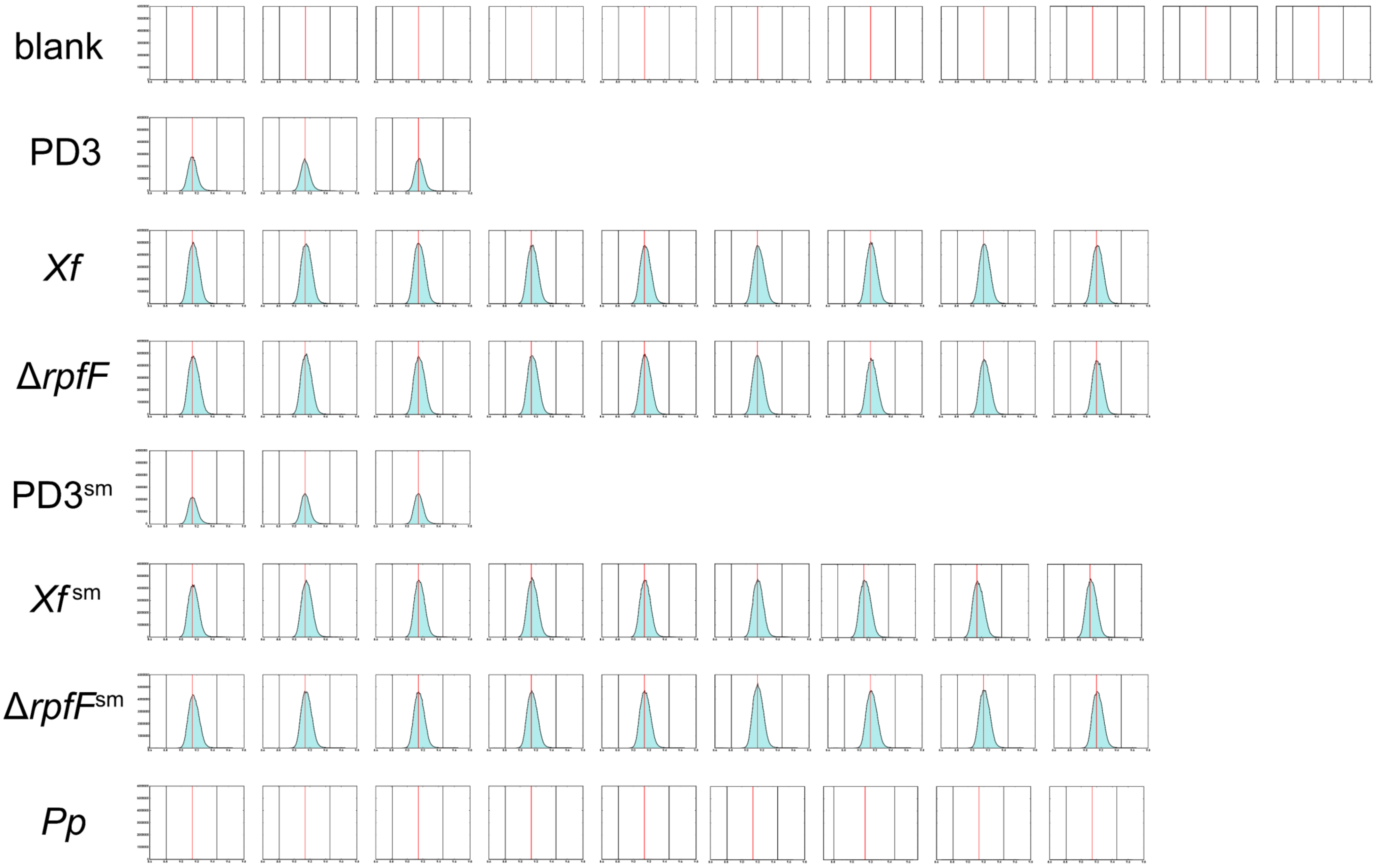

Figure S1F. Phenylalanine. List of EICs from Essential and Non Essential Amino Acids in all conditions and replicates.

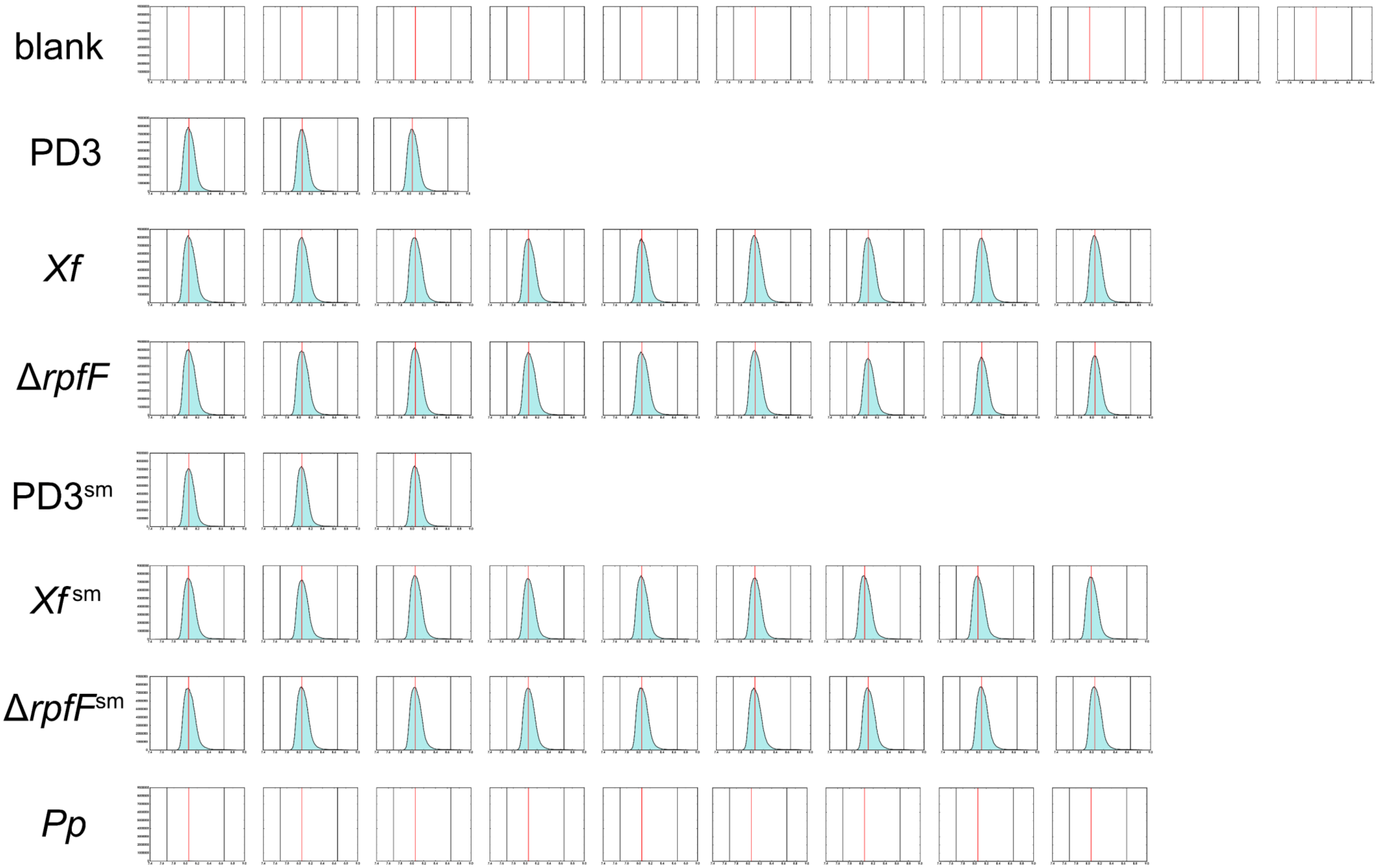

Figure S1G. Arginine. List of EICs from Essential and Non Essential Amino Acids in all conditions and replicates.

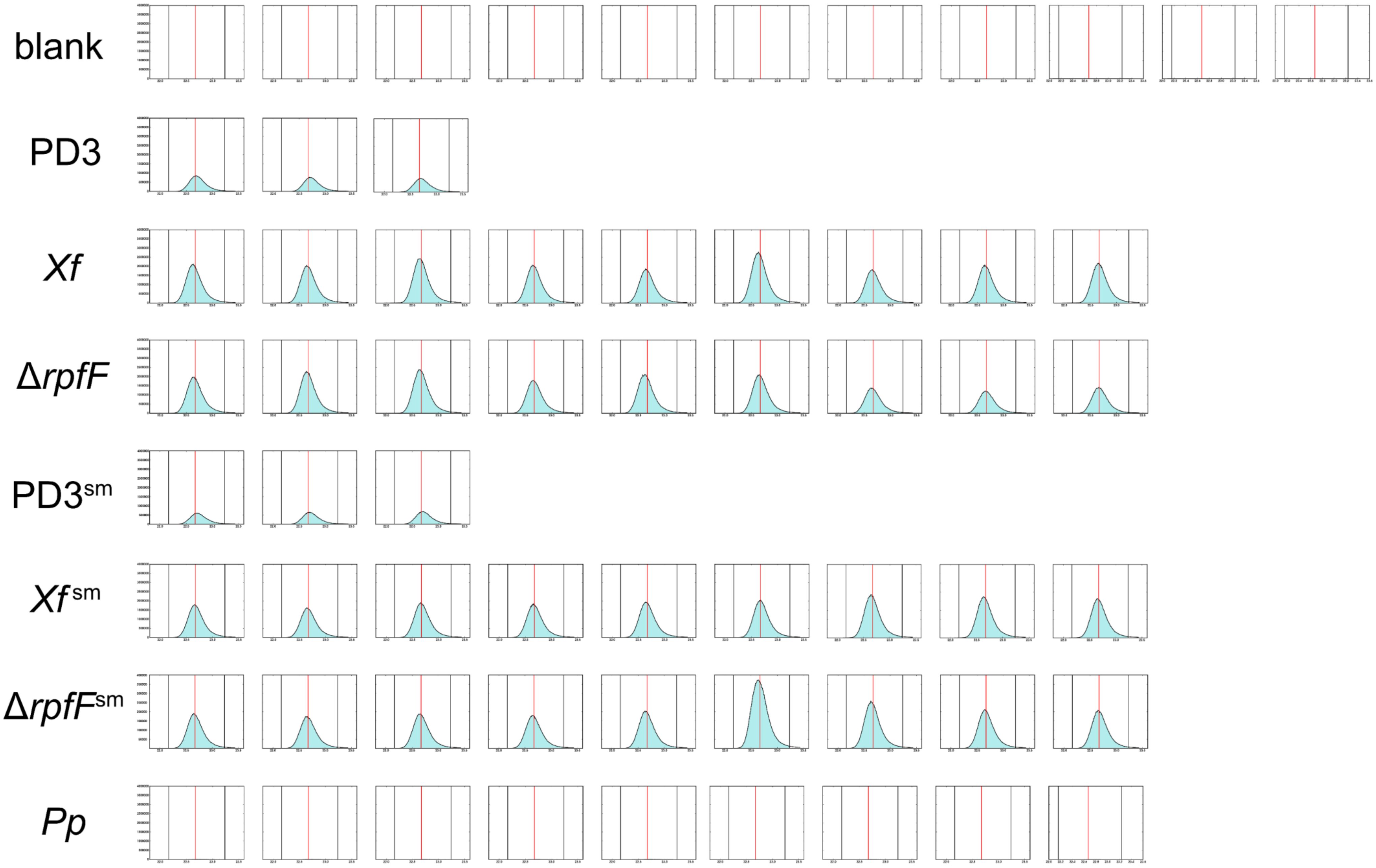

Figure S1H. Isoleucine. List of EICs from Essential and Non Essential Amino Acids in all conditions and replicates.

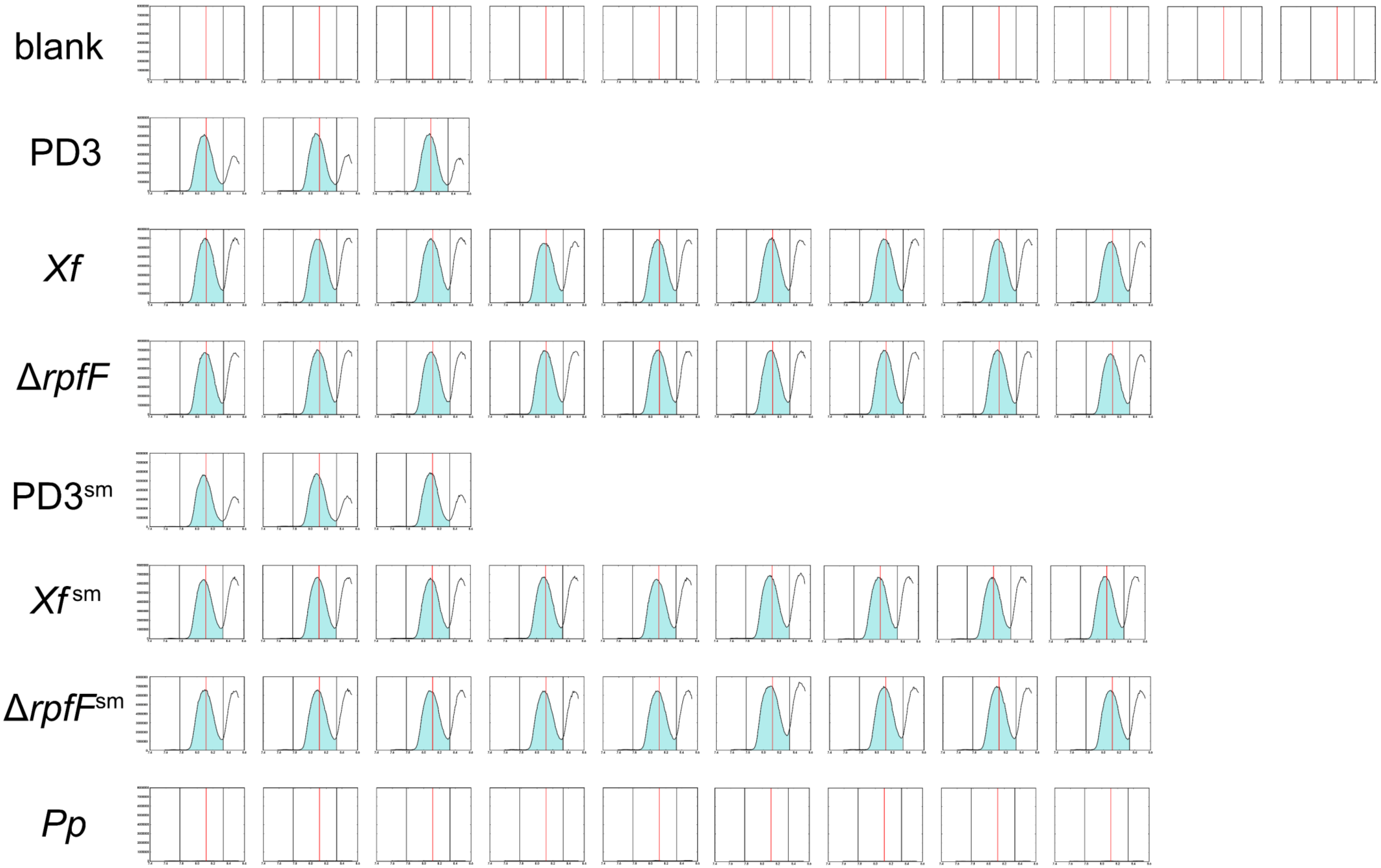

Figure S1I. Leucine. List of EICs from Essential and Non Essential Amino Acids in all conditions and replicates.

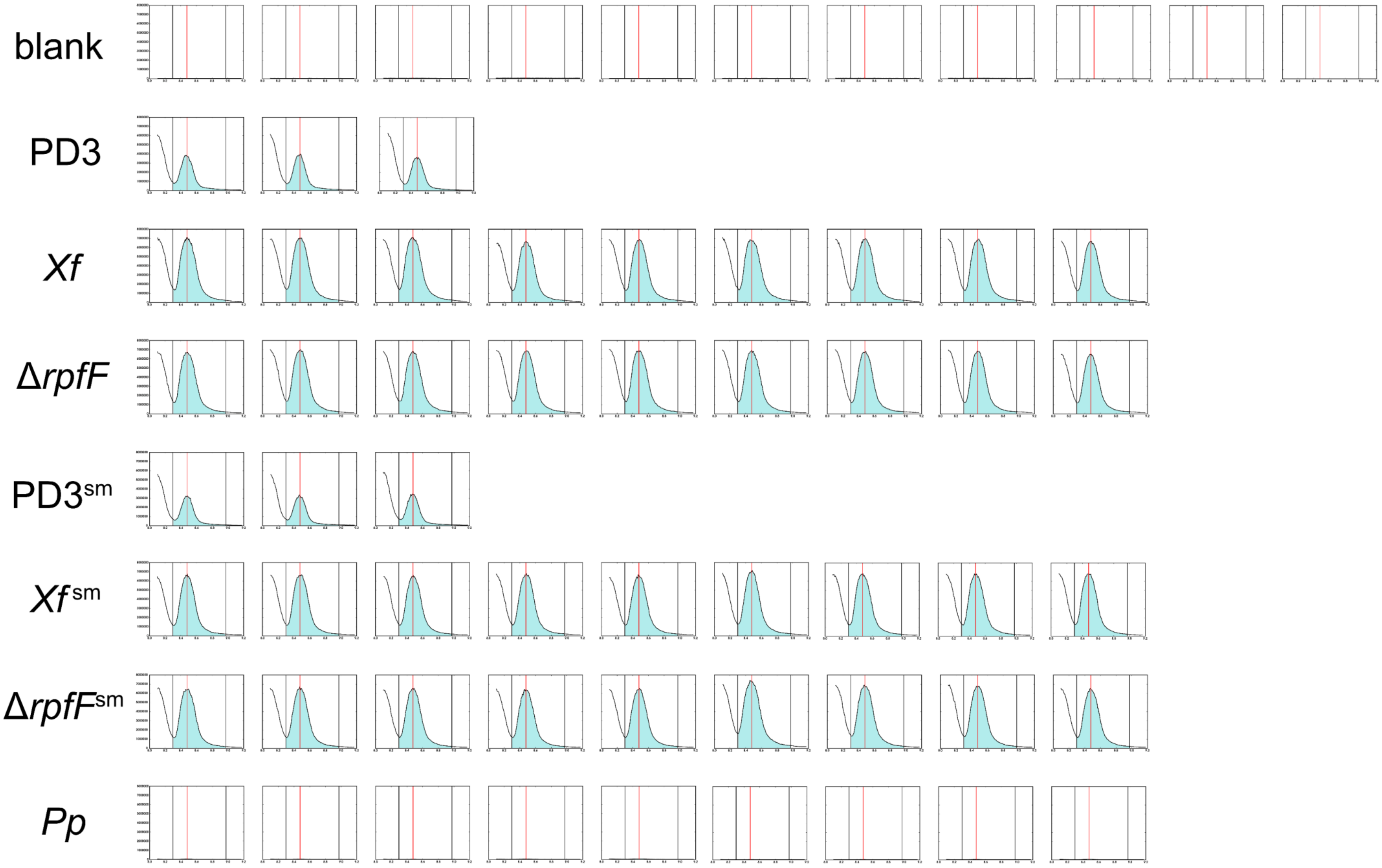

Figure S1J. Threonine. List of EICs from Essential and Non Essential Amino Acids in all conditions and replicates.

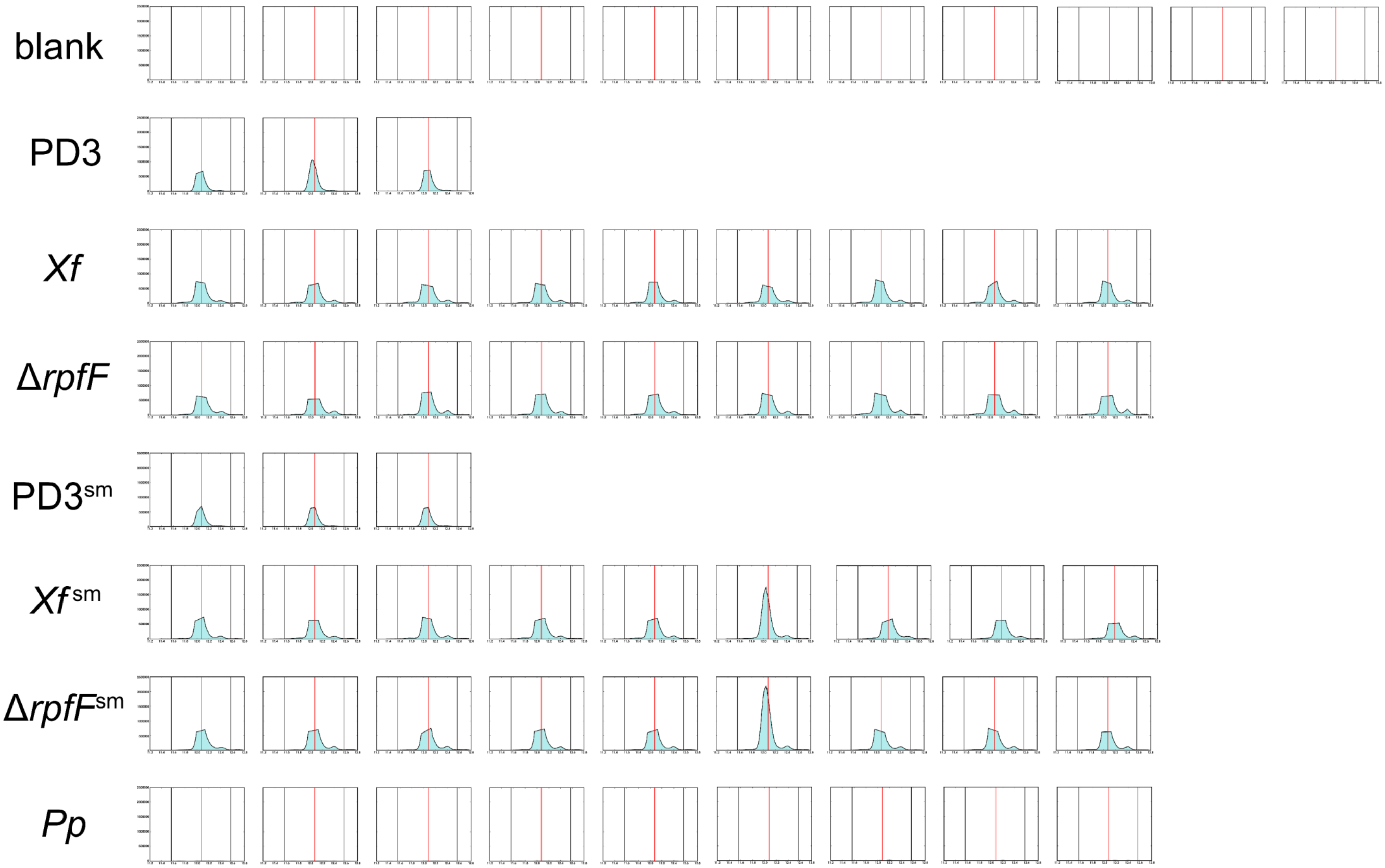

Figure S1K. Cystine. List of EICs from Essential and Non Essential Amino Acids in all conditions and replicates.

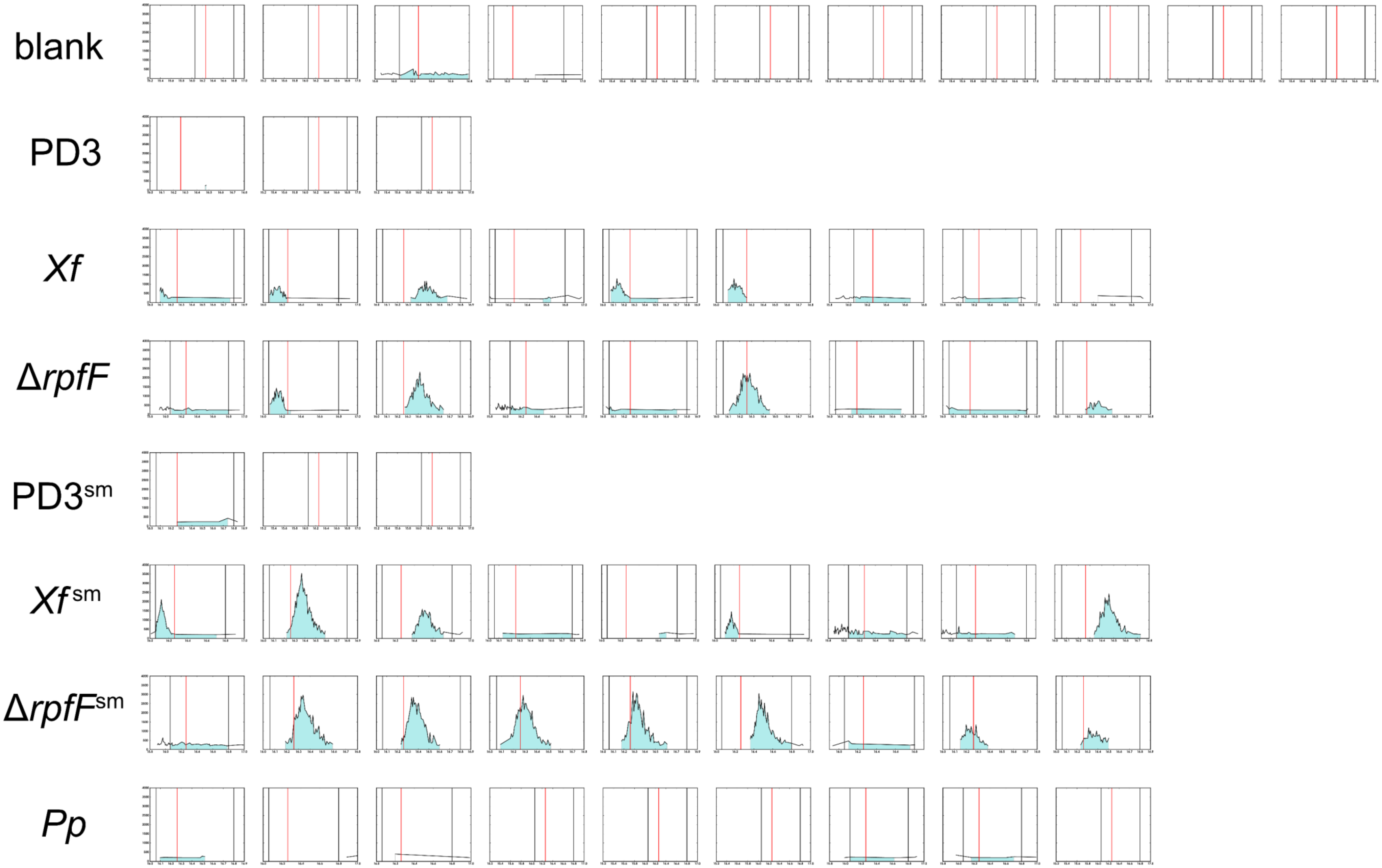

Figure S1L. Aspartate. List of EICs from Essential and Non Essential Amino Acids in all conditions and replicates.

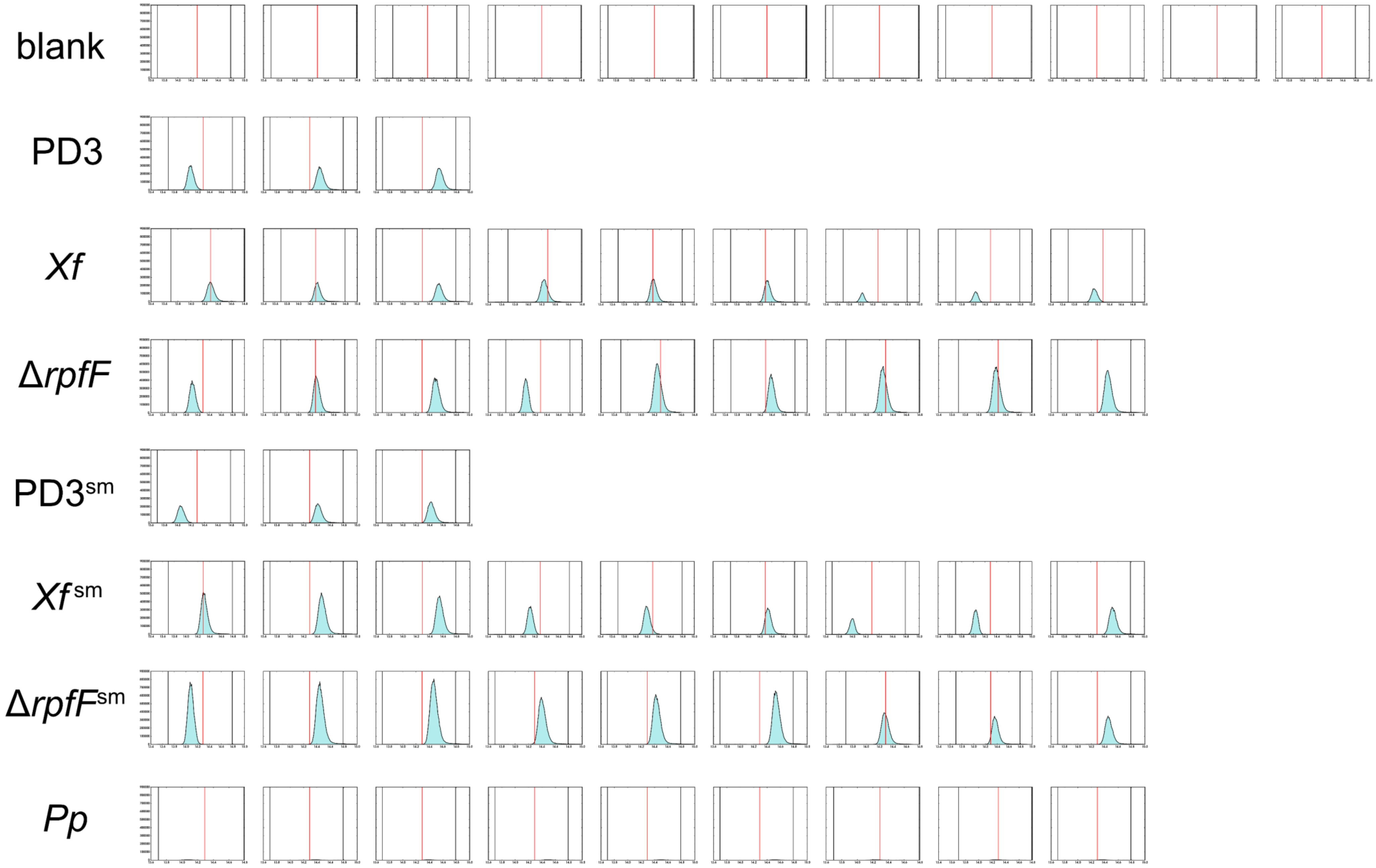

Figure S1M. Glycine. List of EICs from Essential and Non Essential Amino Acids in all conditions and replicates.

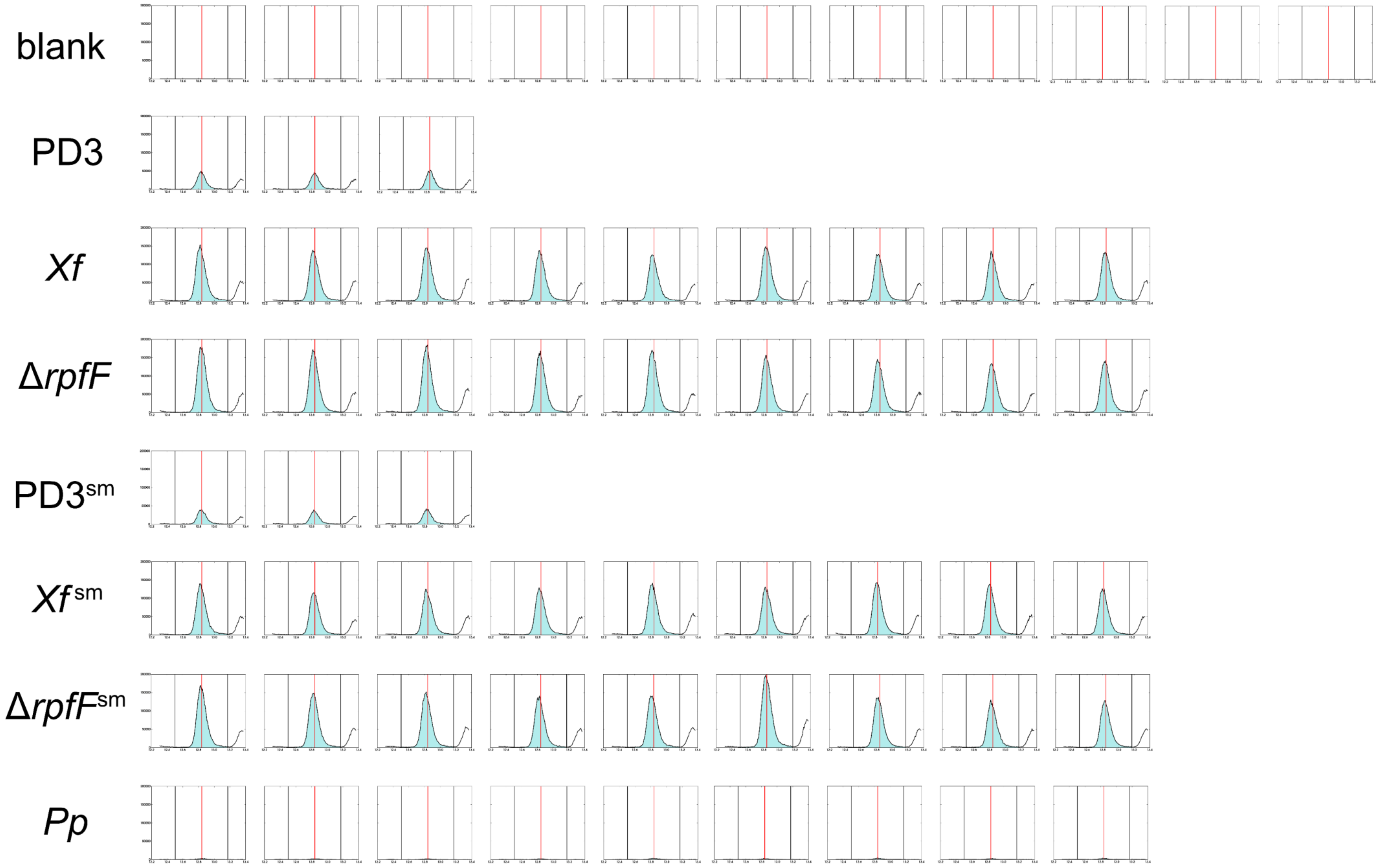

Figure S1N. Serine. List of EICs from Essential and Non Essential Amino Acids in all conditions and replicates.

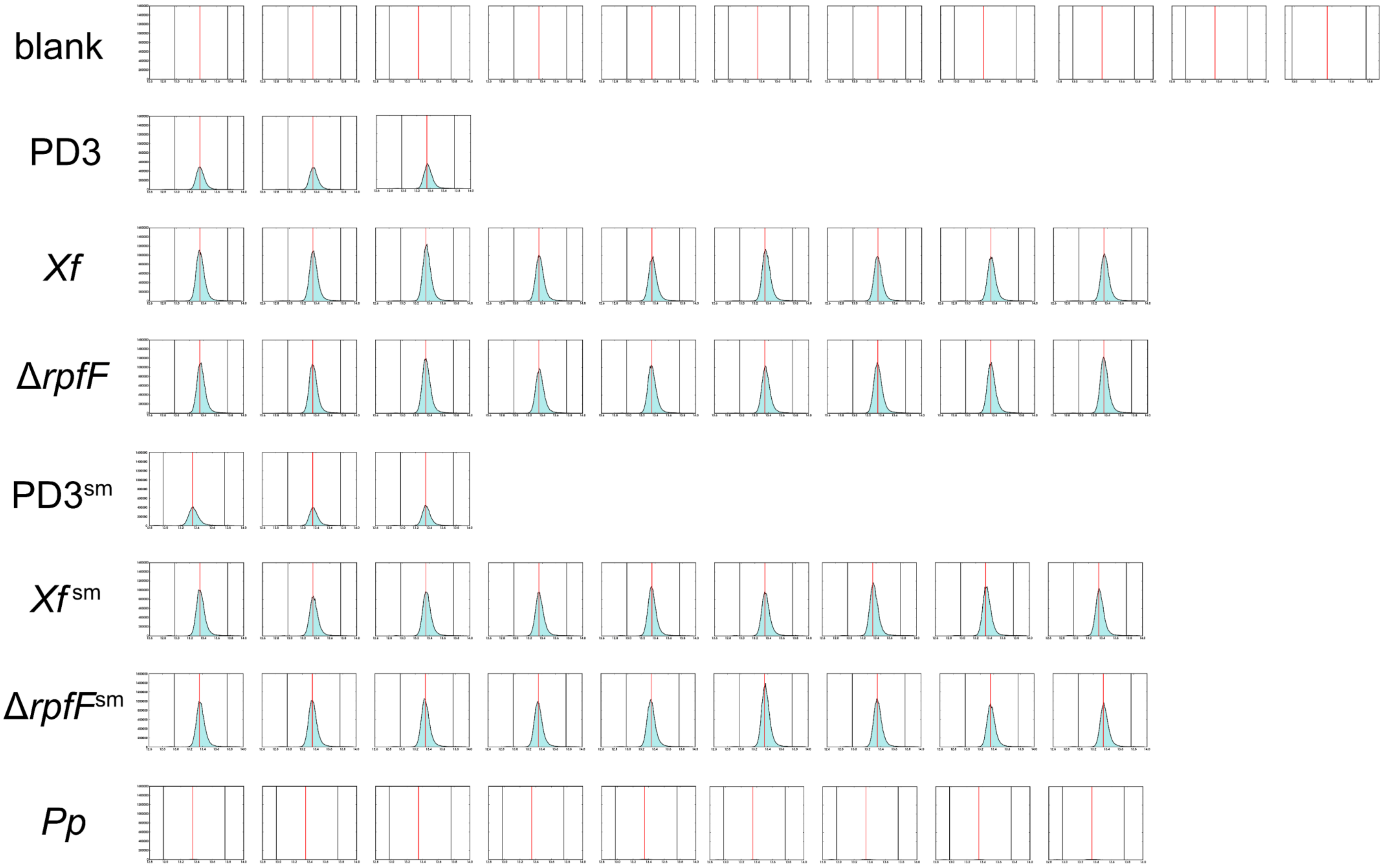

Figure S1O. Glutamate. List of EICs from Essential and Non Essential Amino Acids in all conditions and replicates.

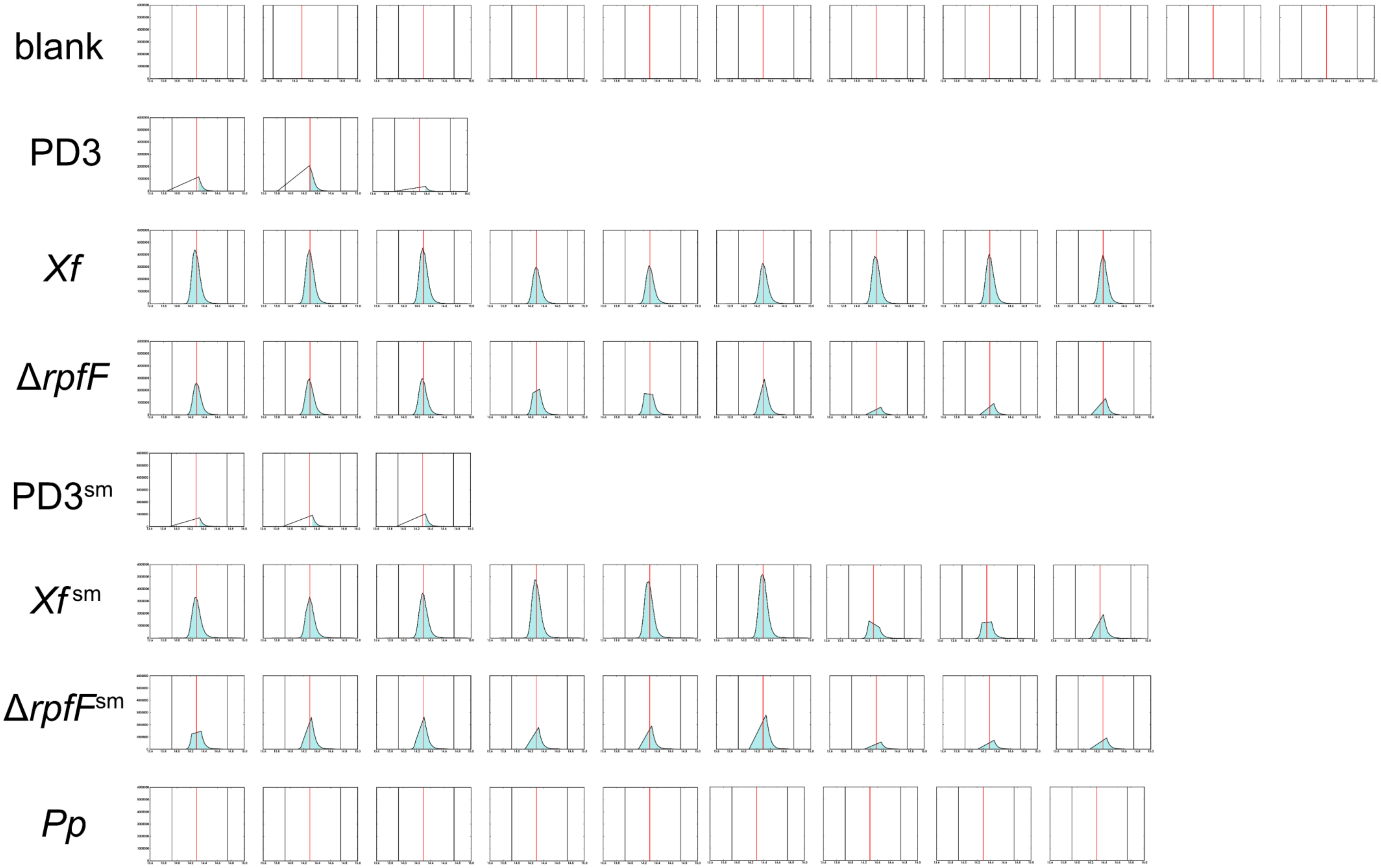

Figure S1P. Alanine. List of EICs from Essential and Non Essential Amino Acids in all conditions and replicates.

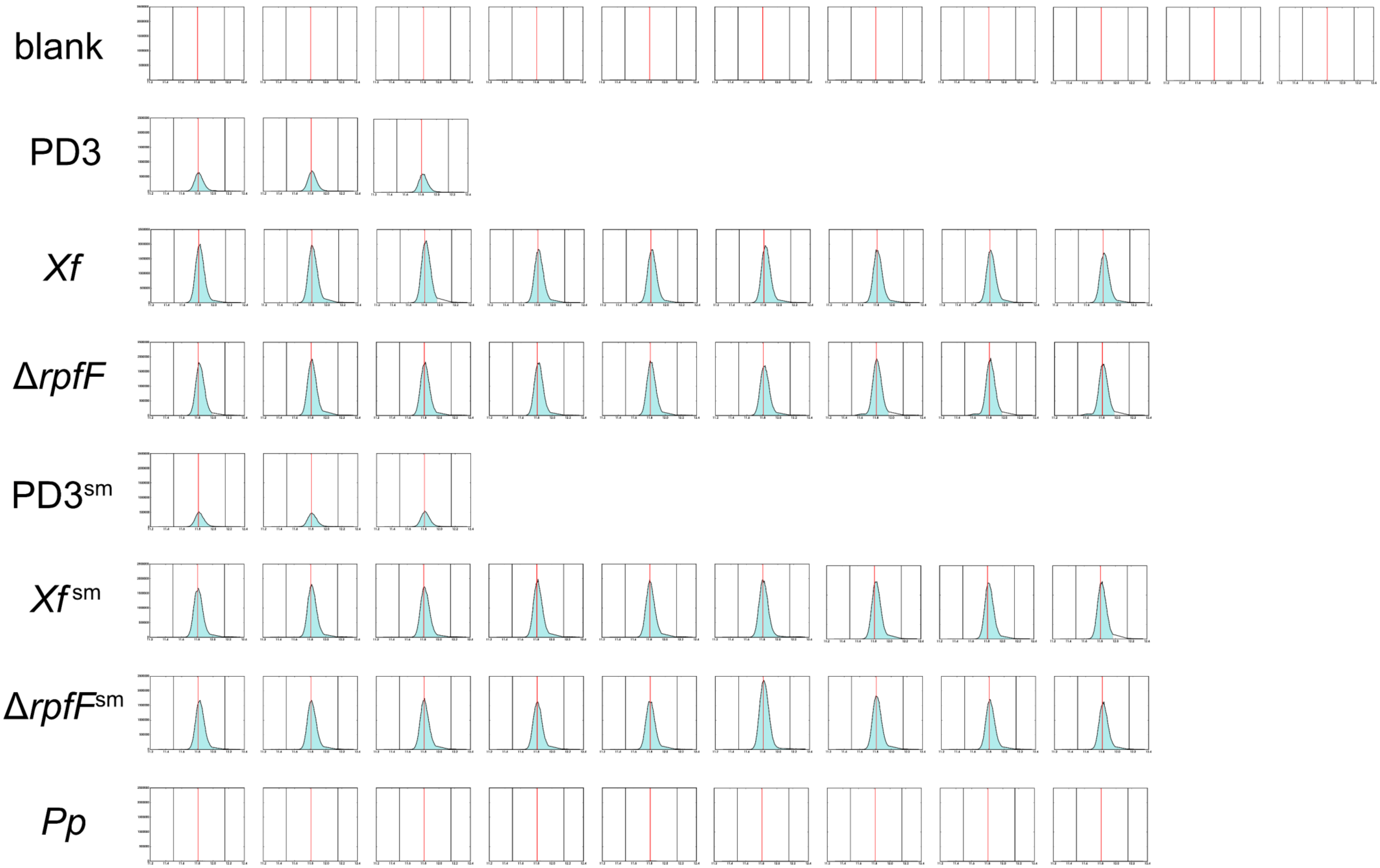

Figure S1Q. Proline. List of EICs from Essential and Non Essential Amino Acids in all conditions and replicates.

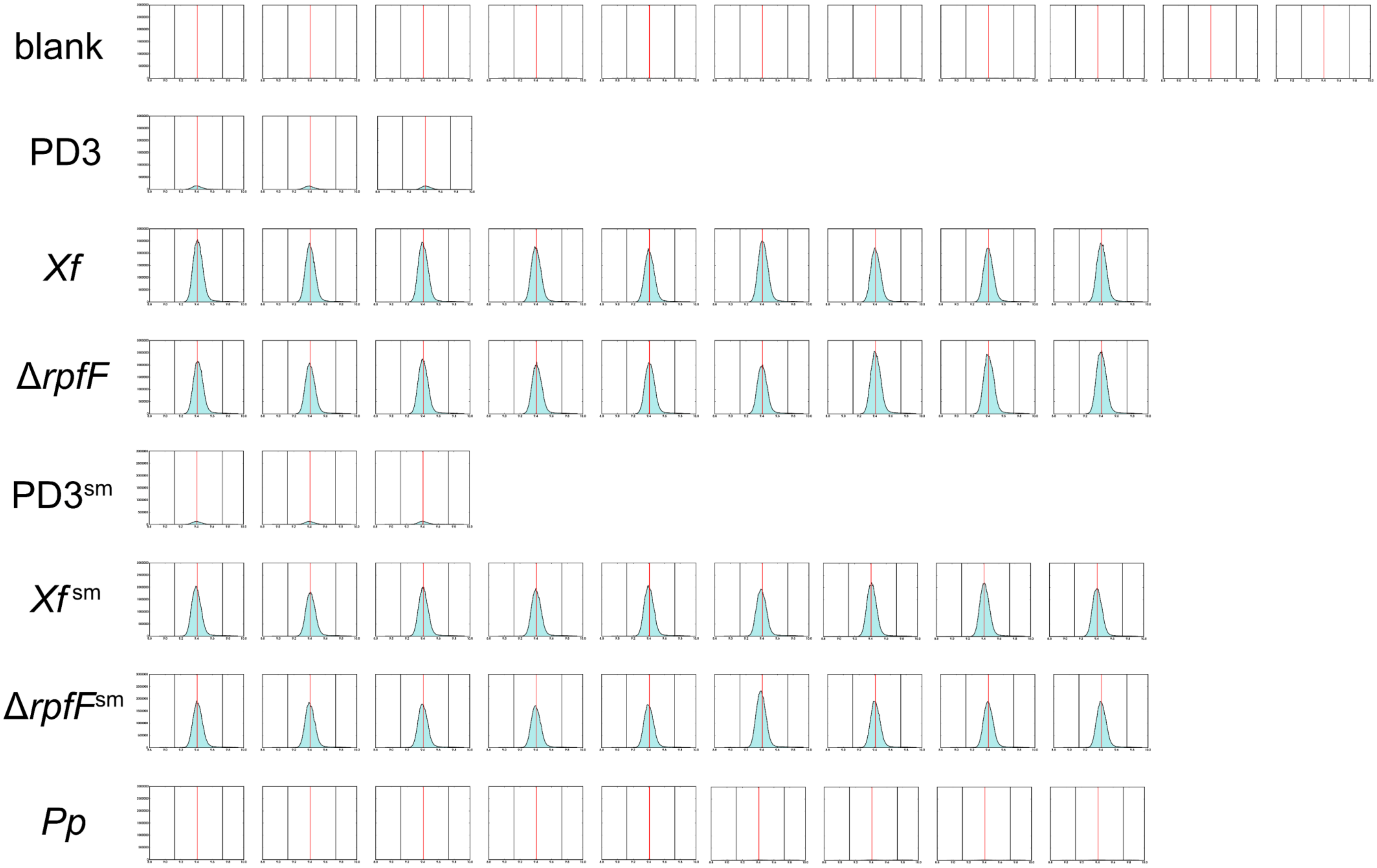

Figure S1R. Asparagine. List of EICs from Essential and Non Essential Amino Acids in all conditions and replicates.

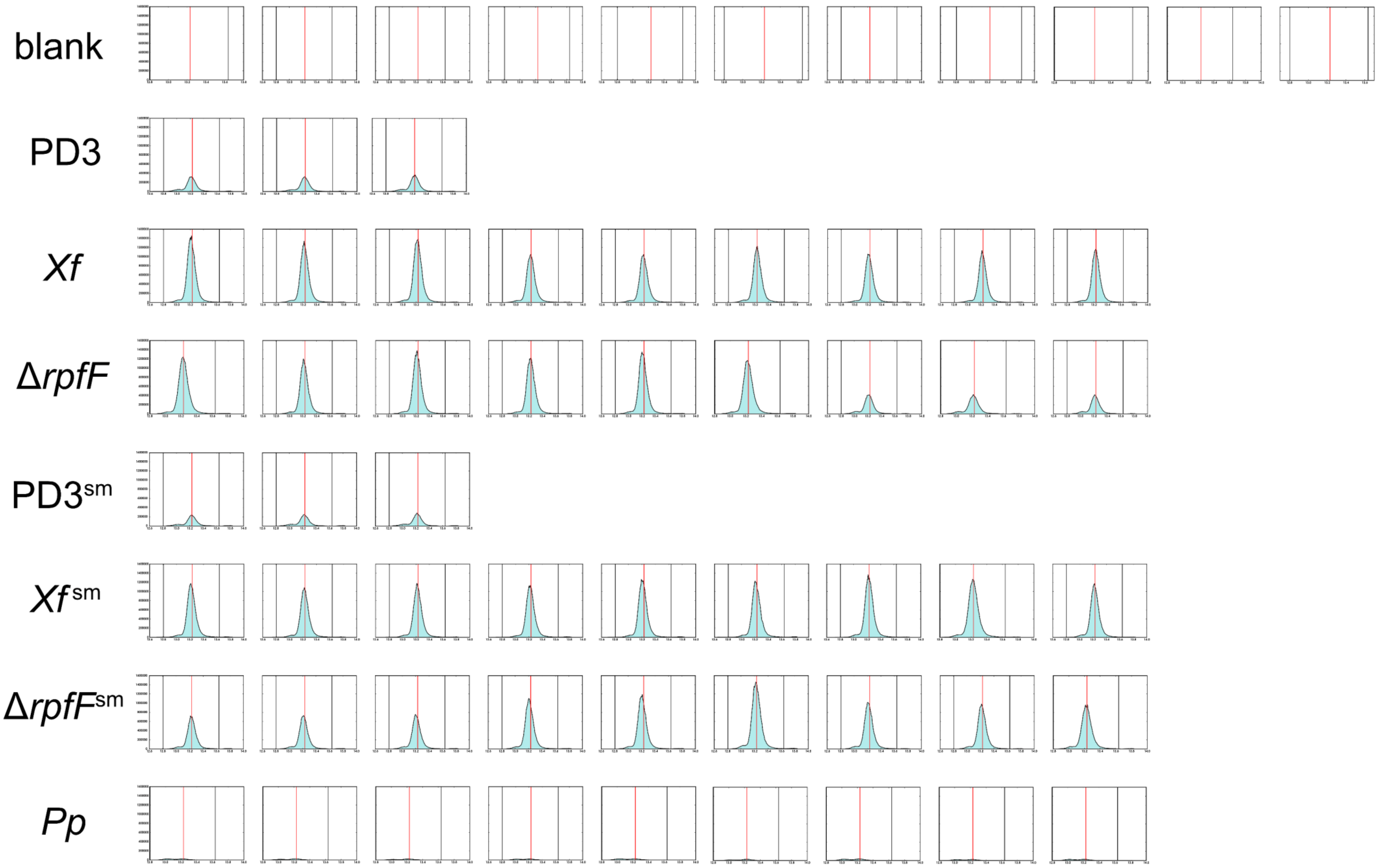

Figure S1S. Tyrosine. List of EICs from Essential and Non Essential Amino Acids in all conditions and replicates.

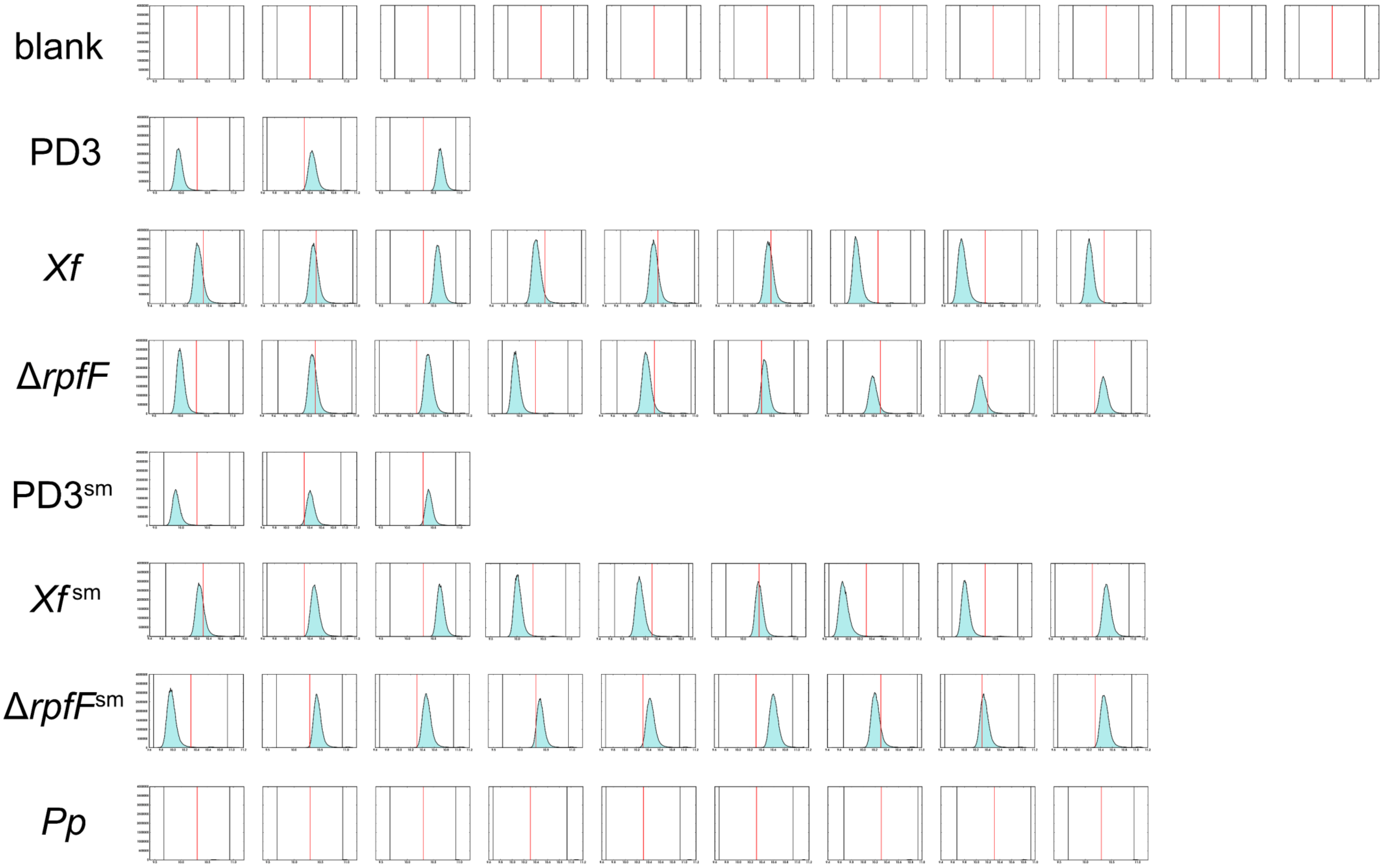

Figure S1T. Glutamine. List of EICs from Essential and Non Essential Amino Acids in all conditions and replicates.

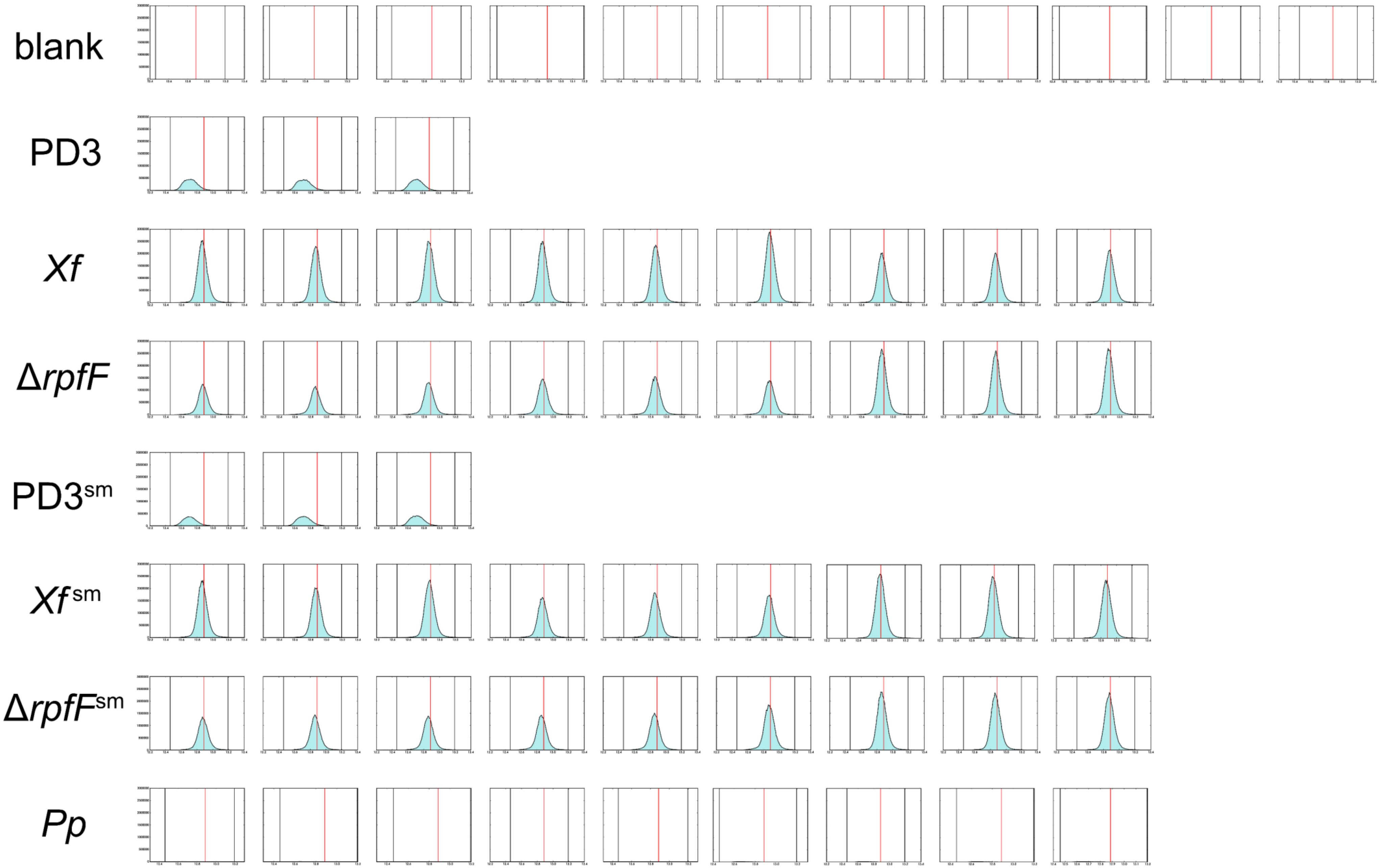

Supplement: Supplementary file 1 [file metabolites-14-00082-s001.zip › Feitosa_et_at_2023_metabolites_Figure_S1.pdf]
