# Supplementary material for: The Exometabolome of Xylella fastidiosa in Contact with Paraburkholderia phytofirmans Supernatant Reveals Changes in Nicotinamide, Amino Acids, Biotin, and Plant Hormones
Source: Metabolites. 2024 Jan 24;14(2):82. doi: 10.3390/metabo14020082 (PMC10890622; doi:10.3390/metabo14020082)

Figure S2A. Nicotinic Acid. List of EICs from Nicotinic Acid, Nicotinamide and Biotin in all conditions and replicates.

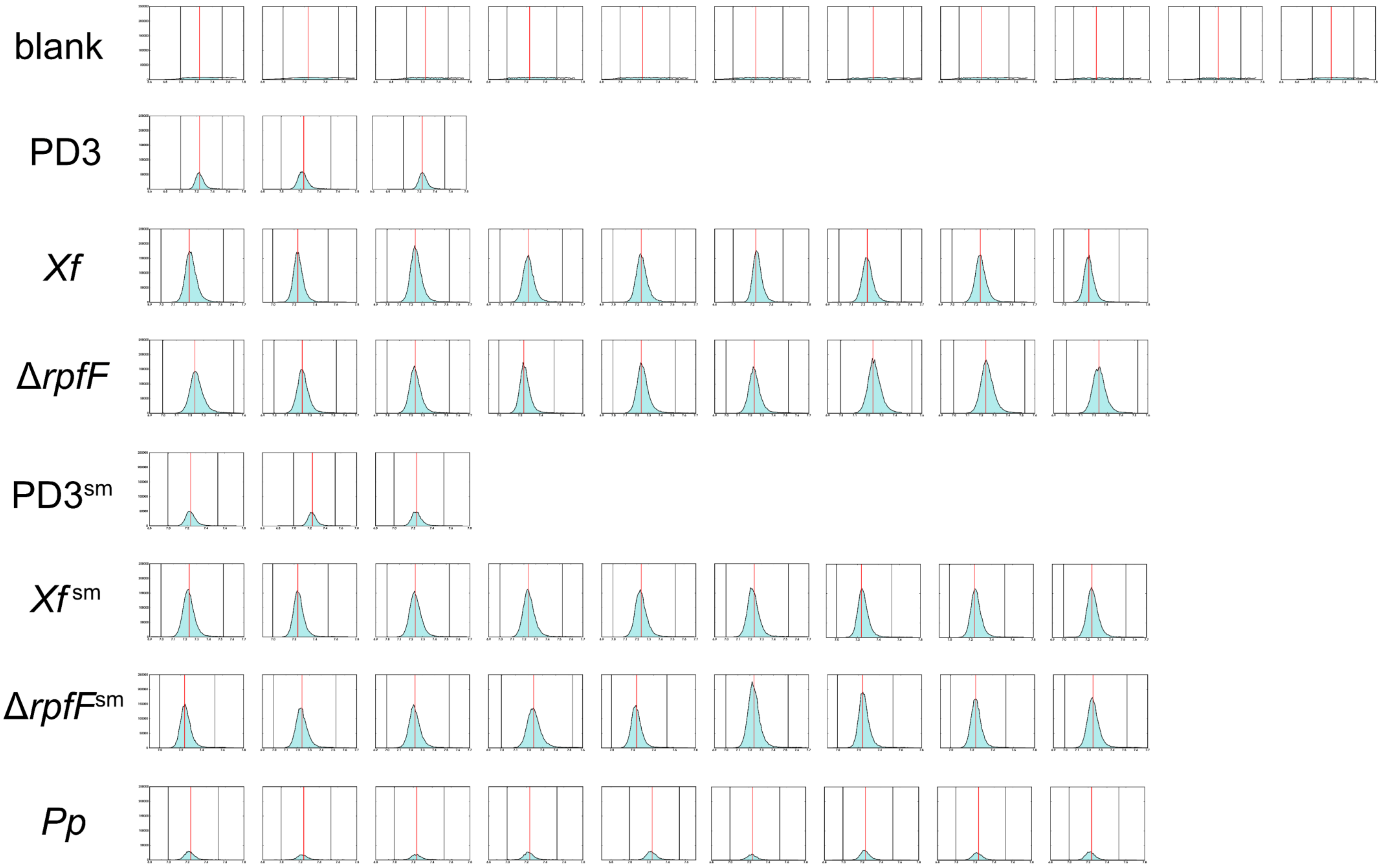

Figure S2B. Nicotinamide. List of EICs from Nicotinic Acid, Nicotinamide and Biotin in all conditions and replicates.

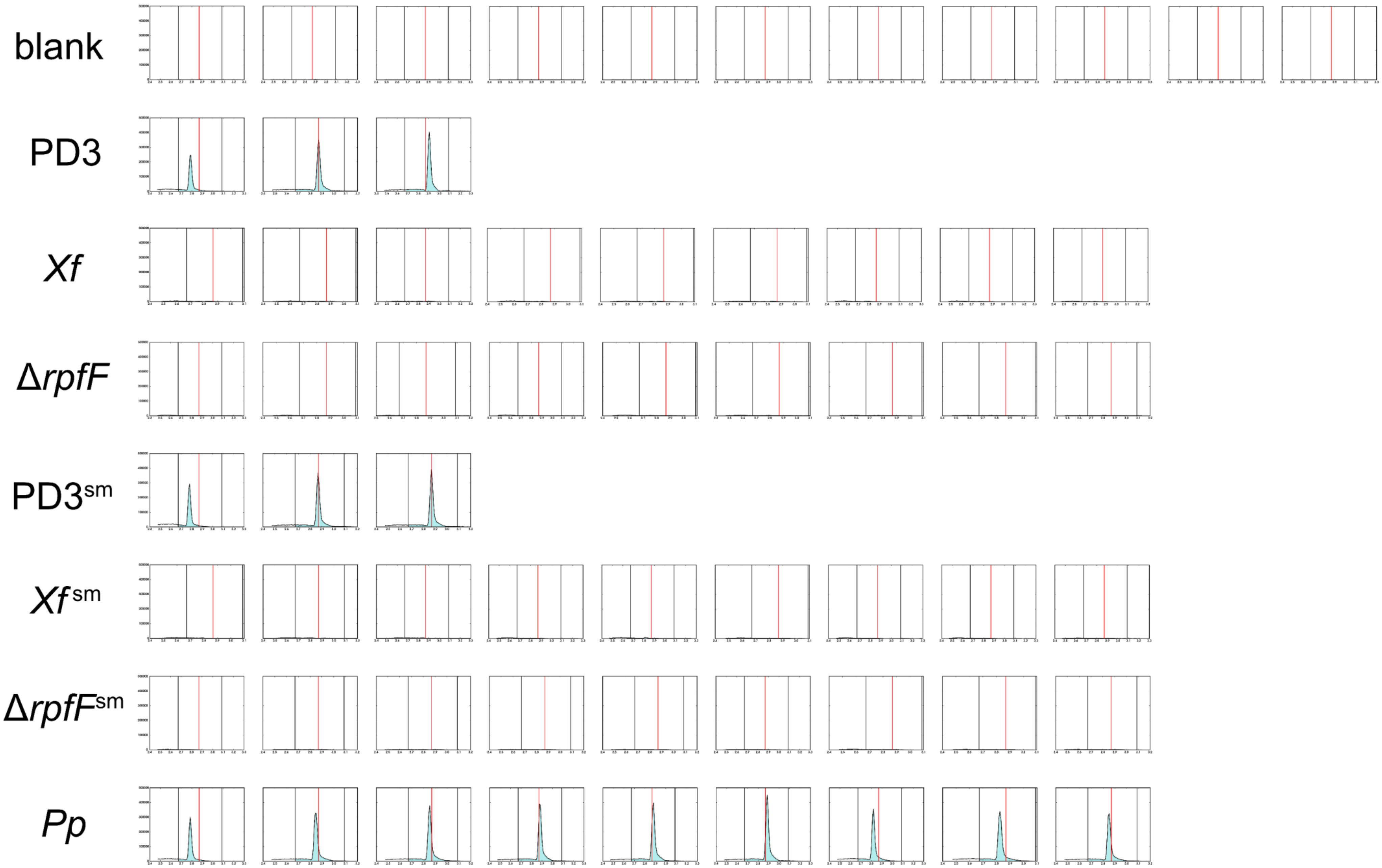

Figure S2C. Biotin. List of EICs from Nicotinic Acid, Nicotinamide and Biotin in all conditions and replicates.

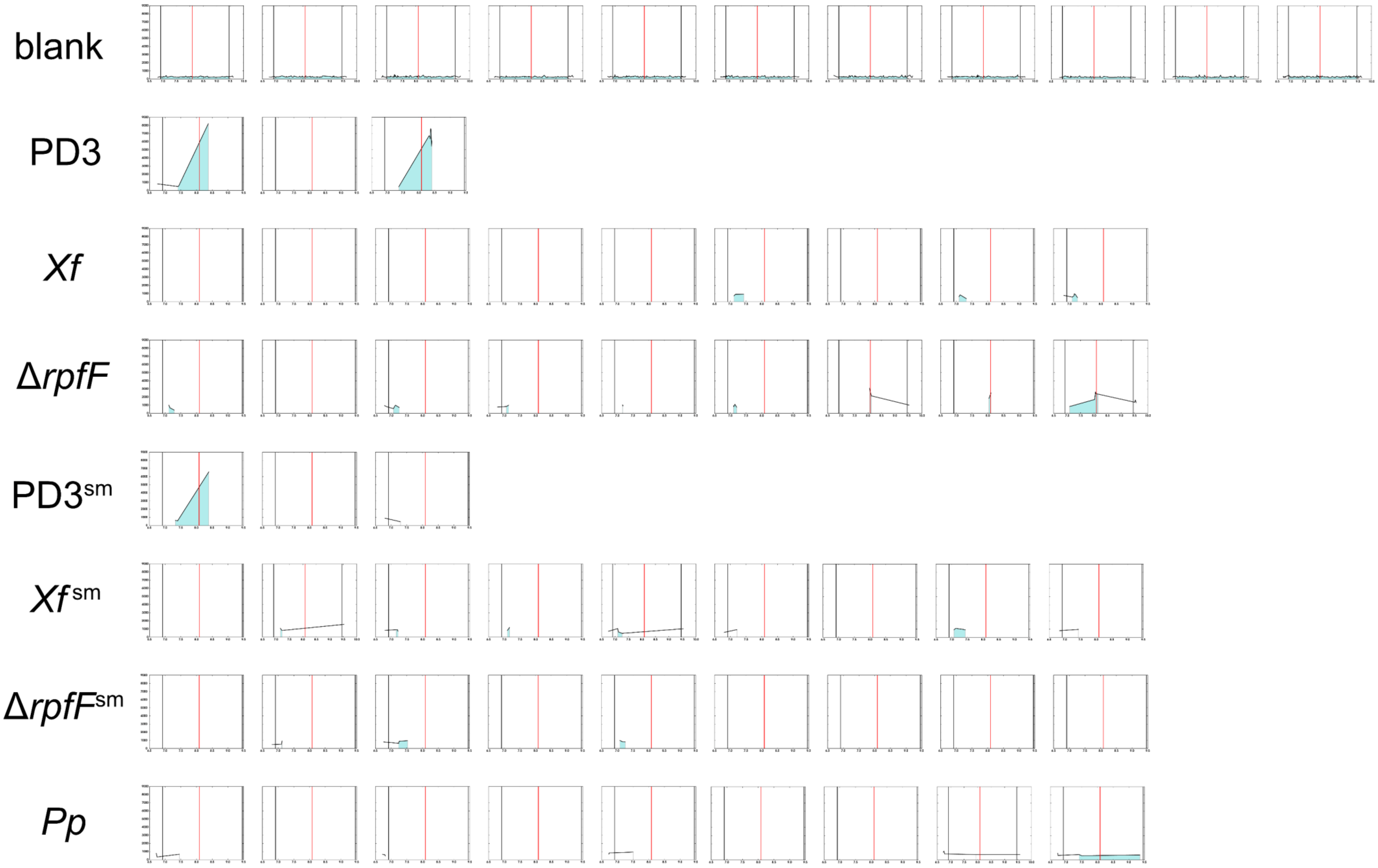

Supplement: Supplementary file 1 [file metabolites-14-00082-s001.zip › Feitosa_et_at_2023_metabolites_Figure_S2.pdf]
