# Supplementary material for: The Exometabolome of Xylella fastidiosa in Contact with Paraburkholderia phytofirmans Supernatant Reveals Changes in Nicotinamide, Amino Acids, Biotin, and Plant Hormones
Source: Metabolites. 2024 Jan 24;14(2):82. doi: 10.3390/metabo14020082 (PMC10890622; doi:10.3390/metabo14020082)

Figure S3A. Isoaminobutyric Acid. List of EICs from Isoaminobutyric Acid and Gibberellic Acid in all conditions and replicates.

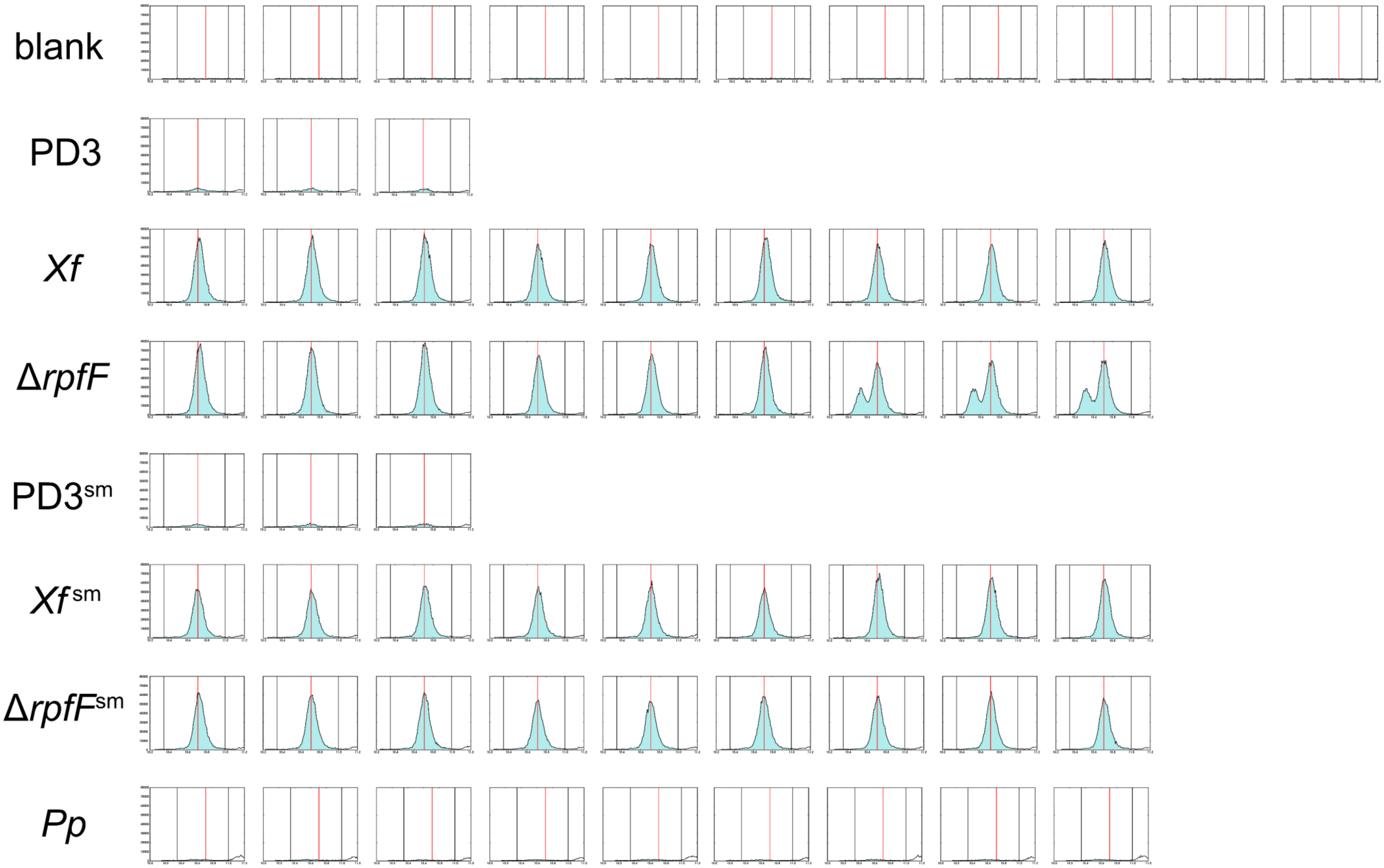

Figure S3B. Gibberellic Acid. List of EICs from Isoaminobutyric Acid and Gibberellic Acid in all conditions and replicates.

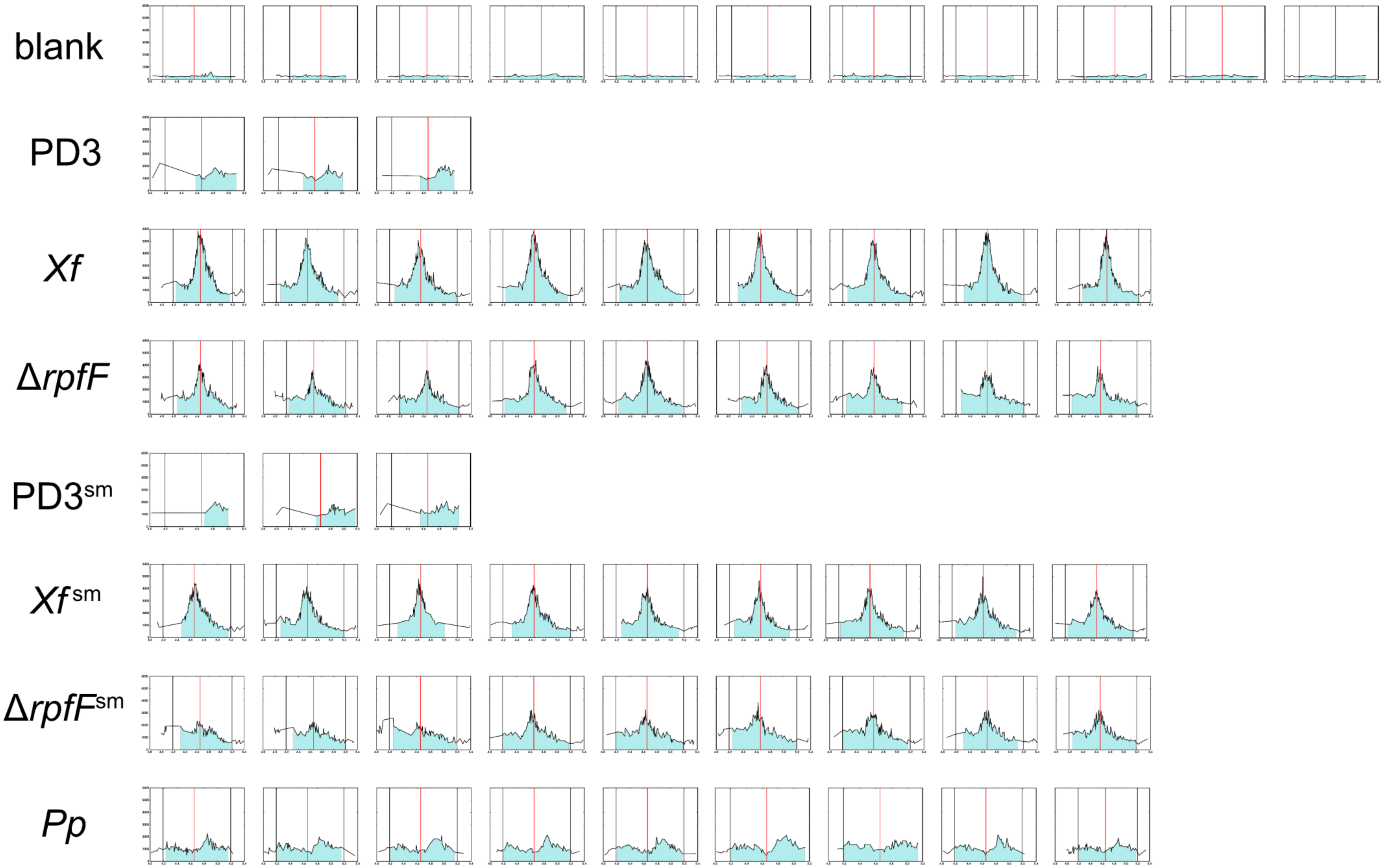

Supplement: Supplementary file 1 [file metabolites-14-00082-s001.zip › Feitosa_et_at_2023_metabolites_Figure_S3.pdf]
