# Supplementary figures and images for: The Exometabolome of Xylella fastidiosa in Contact with Paraburkholderia phytofirmans Supernatant Reveals Changes in Nicotinamide, Amino Acids, Biotin, and Plant Hormones
Source: Metabolites. 2024 Jan 24;14(2):82. doi: 10.3390/metabo14020082 (PMC10890622; doi:10.3390/metabo14020082)

*Paraburkholderia phytofirmans*

NC\_010679.1

NC\_010676.1

NC\_010681.1

NZ\_CP045235.1

NZ\_CP045236.1

NZ\_CP045237.1

*Burkholderia cepacia*

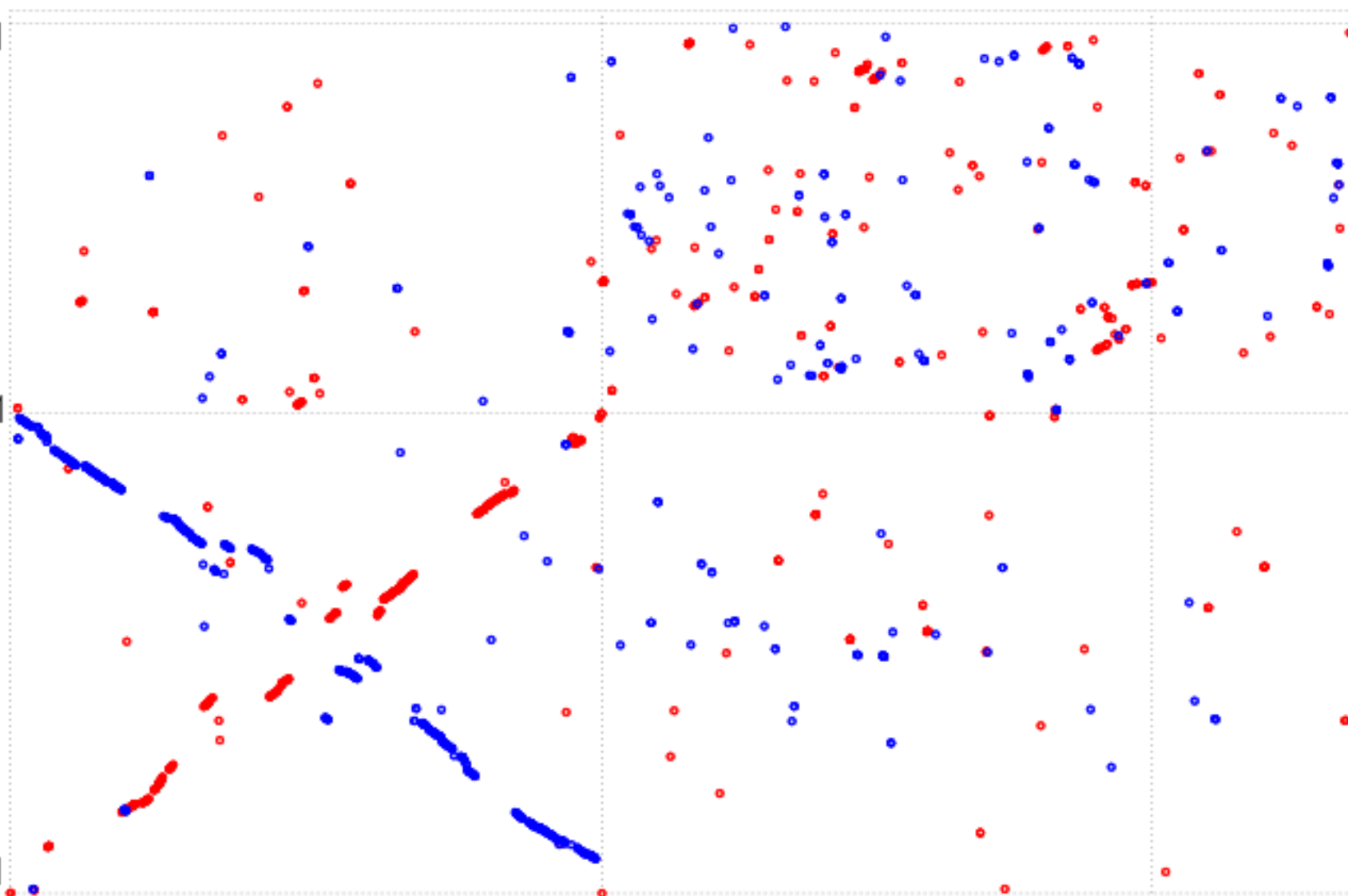

Supplement: Supplementary file 1 [file metabolites-14-00082-s001.zip › Feitosa_et_at_2023_metabolites_Figure_S4.pdf]
